# Combined Effects of Time Spent in Physical Activity, Sedentary Behaviors and Sleep on Obesity and Cardio-Metabolic Health Markers: A Novel Compositional Data Analysis Approach

**DOI:** 10.1371/journal.pone.0139984

**Published:** 2015-10-13

**Authors:** Sebastien F. M. Chastin, Javier Palarea-Albaladejo, Manon L. Dontje, Dawn A. Skelton

**Affiliations:** 1 School of Health and life Science, Institute of Applied Health Research, Glasgow Caledonian University, Glasgow, United Kingdom; 2 Biomathematics and Statistics Scotland, Edinburgh, United Kingdom; Children's National Medical Center, Washington, UNITED STATES

## Abstract

The associations between time spent in sleep, sedentary behaviors (SB) and physical activity with health are usually studied without taking into account that time is finite during the day, so time spent in each of these behaviors are codependent. Therefore, little is known about the combined effect of time spent in sleep, SB and physical activity, that together constitute a composite whole, on obesity and cardio-metabolic health markers. Cross-sectional analysis of NHANES 2005–6 cycle on N = 1937 adults, was undertaken using a compositional analysis paradigm, which accounts for this intrinsic codependence. Time spent in SB, light intensity (LIPA) and moderate to vigorous activity (MVPA) was determined from accelerometry and combined with self-reported sleep time to obtain the 24 hour time budget composition. The distribution of time spent in sleep, SB, LIPA and MVPA is significantly associated with BMI, waist circumference, triglycerides, plasma glucose, plasma insulin (all p<0.001), and systolic (p<0.001) and diastolic blood pressure (p<0.003), but not HDL or LDL. Within the composition, the strongest positive effect is found for the proportion of time spent in MVPA. Strikingly, the effects of MVPA replacing another behavior and of MVPA being displaced by another behavior are asymmetric. For example, re-allocating 10 minutes of SB to MVPA was associated with a lower waist circumference by 0.001% but if 10 minutes of MVPA is displaced by SB this was associated with a 0.84% higher waist circumference. The proportion of time spent in LIPA and SB were detrimentally associated with obesity and cardiovascular disease markers, but the association with SB was stronger. For diabetes risk markers, replacing SB with LIPA was associated with more favorable outcomes. Time spent in MVPA is an important target for intervention and preventing transfer of time from LIPA to SB might lessen the negative effects of physical inactivity.

## Introduction

### Background

The course of a day is made up of a sequence of periods of sleep, sedentary behaviors (SB) such as watching television, light intensity physical activity (LIPA) such as incidental to tasks of daily living, and moderate to vigorous physical activity (MVPA). Research has shown that these behaviors are related to health. For example, there is good evidence that 5 to 7 hours of sleep [1], and over 30 minutes of MVPA per day [2,3] are associated with better health outcomes. More recently, it has emerged that time spent in LIPA might play a positive role in preventing obesity [4] and that sedentary time is detrimental to health [5]. There is some evidence that the influence of SB might be independent of time spent in MVPA [6] but this is ignoring the possible confounding effects of sleep and LIPA. Similarly, time spent in LIPA might have effects independent of time spent on MVPA [7] if the effect of SB and sleep are neglected. To date, the time allocated to each of these behaviors through the day and its relationship to health has been studied in isolation or with only partial adjustment for time spent in other behaviors [8]. However, we know very little about the combined effect of allocating time to these different behaviors that together constitutes a composite whole.

Indeed time is finite, in a circadian or diurnal cycle, and time spent in one behavior necessarily displaces time spent in, at least, another one. Usually, when we monitor daily behavior, either with objective or self-reported instruments, we measure not independent but rather co-dependent relative amounts of time, which together add up to a finite total time equal to either 24 hours, the waking day or wear-time depending on the measurement protocol. Although the physical activity researchers commonly recognize the relative and constrained nature of these data by expressing them in proportions or percentages with respect to the given total, the usual statistical practice fails to account for it. This, for example, causes collinearity problems in multivariate statistical analyses when working with the entire array of behaviors over a fixed total time [8]. Besides, behaviors may not be independent even when they are found uncorrelated (such as time spent in MVPA and SB), as the usual correlation coefficient becomes an inconsistent measure of pair-wise relationships [9]. Inferences about some behaviors should not depend on the presence or absence of any other behaviors in the data. Moreover, conclusions should not depend either on whether the data are expressed in raw units, say minutes, or re-scaled to proportions or percentages. These are all issues that feed current controversies in the epidemiology of daily physical behavior[8,10] and hinder progress. However these issues can be addressed if the fundamental nature of daily time budget as compositional data is acknowledged. Indeed, the distribution of time spent in physical behaviors is by nature intrinsically compositional[11,12].

### Aim

The aim of this study is to investigate the combined effect of time spent in physical activity, sedentary behaviors and sleep that together constitute a composite whole on obesity and cardiometabolic health markers within a compositional data analysis framework. The analysis explores 1) the association between the time budget composition of the day and obesity and cardiometabolic health markers and 2) the consequence on those health markers of displacing time spent in one behavior by another, in order to obtain estimates of the effect of each behavior fully adjusted for time spent in all other behaviors.

## Methods

### Design

This study is a secondary data analysis of the 2005–6 cycle of the National Health and Nutrition Examination Survey (NHANES) study. NHANES is a survey conducted over a two year cycle designed to assess the health and nutritional status of a representative sample of the United States population. The original study was approved by the ethics committee of the Centers for Disease Control and Prevention (CDC) and all participants gave informed consent. Description of the method and procedures used in NHANES are detailed on the CDC website[13]. This analysis focuses only on the 2005–6 cycle as this is the only cycle that included concurrent waking day assessment of activity via accelerometry and self-reported data on sleep duration. The NHANES dataset was chosen because it is a well characterized data set which has been used extensively to determine the association of daily time spent sleeping, sedentary and in light and moderate to vigorous physical activity with cardiometabolic health markers [1,6,10,14–17]. Therefore it provides a direct means of comparison of the results obtained using compositional analysis with those derived from standard statistical methods.

### Participants

A sample of adult (21 to 64 years old inclusive) participants was drawn from the 2005–6 NHANES cycle. Criteria for inclusion in this analysis included 5 days of valid accelerometry data according to the CDC accelerometry criteria [15,17]. Participants with missing self-reported sleep, covariates and biomarker data were excluded. There were 3688 adults eligible to wear an accelerometer in the total cycle sample of 10348 participants of all ages. Amongst those, data from 1937 were available for analysis. Further details about the sample are provided in supplementary material (Table A and Fig A in S1 File).

### Assessment of composition of the day

The day was partitioned in proportion of time spent in four physical behaviors: sleep, sedentary behaviors (SB), light intensity physical activity (LIPA) and moderate to vigorous physical activity (MVPA).

Time spent in SB, LIPA and MVPA was assessed objectively following the protocol detailed previously [15,17], using an accelerometer (Actigraph 7164; Actigraph, LLC, Pensacola, FL). This device was worn on the hip for seven days during waking hours. These data, which consist of acceleration counts integrated over 1 minute epochs, were processed according to standard quality assurance procedures [15,17]. Days when the accelerometer was worn for at least 10 hours were considered valid and participants were included if at least 5 valid days of their activity were available. Each minute epoch was classified using standard count per minutes thresholds as SB (<100 counts/min), LIPA (100 to 1951 counts/min) or MVPA (≥ 1952 counts/min) [18]. Minutes spent in each of these three behaviors were tallied per day and averaged over all available valid days, expressed as proportions of 24 hours.

Sleep duration was self-reported as an integer from 1 to 24 hours in response to the question “How much sleep do you actually get at night on weekdays or workdays”. Sleep time was then also expressed as a proportion of 24 hours.

The proportion of 24 hours spent in sleep, SB, LIPA and MVPA were normalized for each participant so that their sum equalled one. The normalization procedure is detailed in S2 File.

### Cardiometabolic markers

Participants attended a mobile examination center where their height and weight was measured for the calculation of body mass index (BMI) and their waist circumference (WC) and blood pressure recorded. Blood samples from the participants were analyzed for non-fasting high-density lipoprotein (HDL) cholesterol and C-reactive protein (CRP). A sub-sample also provided fasting specimen for measurement of fasting low-density-lipoprotein (LDL) cholesterol, triglycerides (TRI), plasma glucose (GLU) and insulin (INS). The latter two were used to compute homeostasis model assessment of insulin resistance (HOMA) using standard methods. Details of these procedure can be found elsewhere [13].

### Socio-demographic data and covariates

Potential confounding factors were extracted from self-reported questionnaires. These included socio-demographic variables; age, sex, race/ethnicity (Mexican American, other Hispanic, non-Hispanic white, non-Hispanic black, or other/multi-racial); marital status (married/living together, widowed, divorced, separated, or never married); education (<12 years, 12 years or equivalent, or some college or above); work status (not working, working part-time (<35 hours/week), or working full-time (≥35 hours/week); and ratio of family income to poverty level. Lifestyle factors considered were smoking status, consumption of caffeine and alcohol and total energy and saturated fat dietary intake. Self-reported health (SF-12), previous diagnosis of cancer, diabetes, cardiovascular disease and stroke diabetes; and current use of diabetic, antihypertensive or lipidemic drugs were also extracted.

### Data analysis

The analysis conducted is based on a compositional data paradigm. Compositional data analysis is currently a mature and well-established field of statistics which has been used in diverse fields of research concerned with multivariate proportion-type data such as nutrition (e.g. fatty acid composition in meat) [19], geochemistry (e.g. sedimentary composition of rocks or chemistry of groundwaters) [20, 21], politics (e.g. multiparty electoral data) [22], and behavioral biology (e.g. fish mating preferences) [23].

Standard and compositional descriptive statistics were computed for comparison. As an alternative to the usual arithmetic mean, the compositional mean or center is obtained by, firstly, computing the geometric mean for each behavior separately and then normalizing the data to the same constant as the raw data, typically 1 or 100. This measure is coherent with the relative and symmetric scale of the data and has been shown to be a better representative of the center of a cloud of compositional data points [11]. Moreover, univariate statistical measures of dispersion, as commonly measured by the standard deviation, are not coherent with the intrinsically inter-dependent multivariate nature of compositional data. The univariate variance of a compositional variable actually contains no information as the variability of the time spent on a single behavior is necessarily linked to the variability of the time spent in another one [24]. Instead, the dispersion in compositional data sets is properly estimated using the variation matrix [11]. This summarizes the variability structure of a data by means of log-ratio variances, that is, variances of the logs of all pair-wise ratios between behaviors.

Linear regression models were fitted to examine the associations between cardiometabolic risk markers and time spent in sleep, SB, LIPA and MVPA. We conducted two separate analyses: (1) firstly, a set of four models using standard regression analysis with the proportion of time spent in a single behavior as exposure variable (SINGLE) were fitted for each outcome to estimate the univariate association between each behavior and the outcome. These models do not adjust for time spent in the other behaviors and do not consider the effect of displacement of time from one activity to another one; (2) secondly, we adopted a compositional approach (CODA) based on an isometric log-ratio (ilr) data transformation adapted from [25] (the method is detailed step by step in S2 File) to adequately adjust the models for time spent in the other behaviors. The log-ratio methodology allows the use of standard statistical methods on the transformed data and then to translate back results into the original units. In the CODA regression models, the entire composition of the daily time spent in all four behaviors acts as exposure variable. These models were used to estimate: (a) the association of each behavior with the outcome adjusted for the time spent in all and each of the other behaviors, (b) the combined effect of the relative distribution of all the behaviors and (c) the effect of displacing time from one behavior to another one.

In all models, non-normally distributed outcome variables were log-transformed. Confounders were entered in the models as covariates by backward elimination and were retained if the corresponding p-values were <0.2. The same set of confounders was adopted in the SINGLE and CODA regression models for each outcome (Table B in S1 File). The linearity of the association between predictors and outcome, as well as the usual requirements for the model residuals, was examined. In accordance with STROBE guidelines, a sensitivity analysis [26] was conducted for each model by removing 10% of cases at random and checking for a statistically significant change in the results. Statistical evidence of association was concluded for test p-values below the usual 0.05 significance level. All analyses were conducted using the R statistical system version 3.1.1.

## Results

### Descriptive statistics

Descriptive statistics of the proportion of time spent in the four behaviors obtained via standard and compositional statistics are displayed in Table 1. The most obvious difference is found with the mean relative amount of time spent in MVPA, which is over-estimated by the arithmetic mean with respect to the compositional alternative by almost 0.7% of a day, roughly 10 minutes.

**Table 1:** Standard and compositional descriptive measures of the proportion of time spent in sleep, sedentary behaviors (SB), light activity (LIPA) and moderate to vigorous activity (MVPA): arithmetic mean (plus standard deviation in parenthesis) and compositional mean. Results expressed in percentage of 24 hours (see text for details).

	Sleep	SB	LIPA	MVPA
Arithmetic mean	28.31 (5.62)	40.21 (9.06)	29.32(8.01)	2.16 (1.99)
Compositional mean	28.71	40.58	29.23	1.48

The variability of the data is summarized in the variation matrix (Table 2) containing all pair-wise log-ratio variances. A value close to zero implies that the times spent in the two behaviors involved in the ratio (arranged by rows and columns) are highly proportional. For example, the variance of log(sleep/SB) is 0.148, which reflects the highest (proportional) relationship or co-dependence (not correlation in the usual sense) between two behaviors. On the other end, it can be observed that the highest log-ratio variances all involve MVPA, which shows that time spent in MVPA is the least co-dependent on the other behaviors. This could explain why the effect of other behaviors can appear spuriously independent of MVPA using standard statistics.

**Table 2:** Compositional variation matrix of time spent in sleep, sedentary behaviors (SB), light activity (LIPA), and moderate to vigorous activity (MVPA).

	Sleep	SB	LIPA	MVPA
Sleep	0	0.148	0.168	1.077
SB	0.148	0	0.248	1.285
LIPA	0.168	0.248	0	0.909
MVPA	1.077	1.285	0.909	0

**Fig 1:**
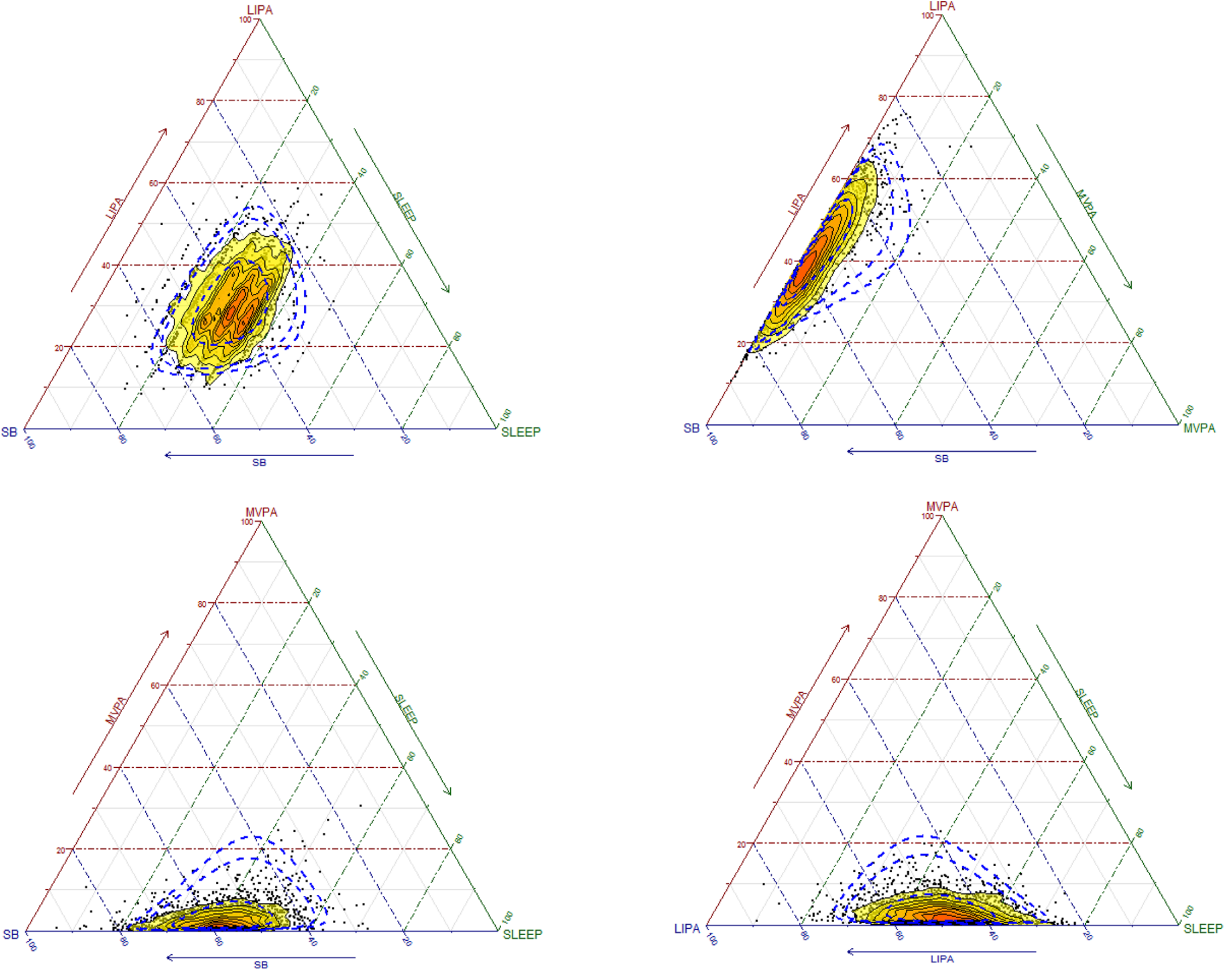
Ternary plots of the sample compositions of time spent in sleep, sedentary behaviors (SB), light activity (LIPA) and moderate to vigorous activity (MVPA) (black dots). The overlapped heat map represents the distribution of the data points (the more intense the color the higher the concentration of data points). The dotted lines refer to 95% and 99% normal-based probability regions.

The distribution of the sample compositions is shown in Fig 1 by means of a matrix of ternary plots with three behaviors represented at a time. Ternary plots can be understood as the scatterplots of compositions (see S2 File for more details). Note that plotting the four behaviors together is possible but would require a 3-dimensional pyramidal plot, which may be difficult to visualize. The overlapped heat map allows distinguishing the areas of highest (more intense color) and lowest (less intense color) data concentration. The dispersion structure is represented by 99% and 95% normal-based probability regions around the compositional center. These reflect that the highest variability is found in the direction of MVPA.

### Composition of the day by groups

**Fig 2:**
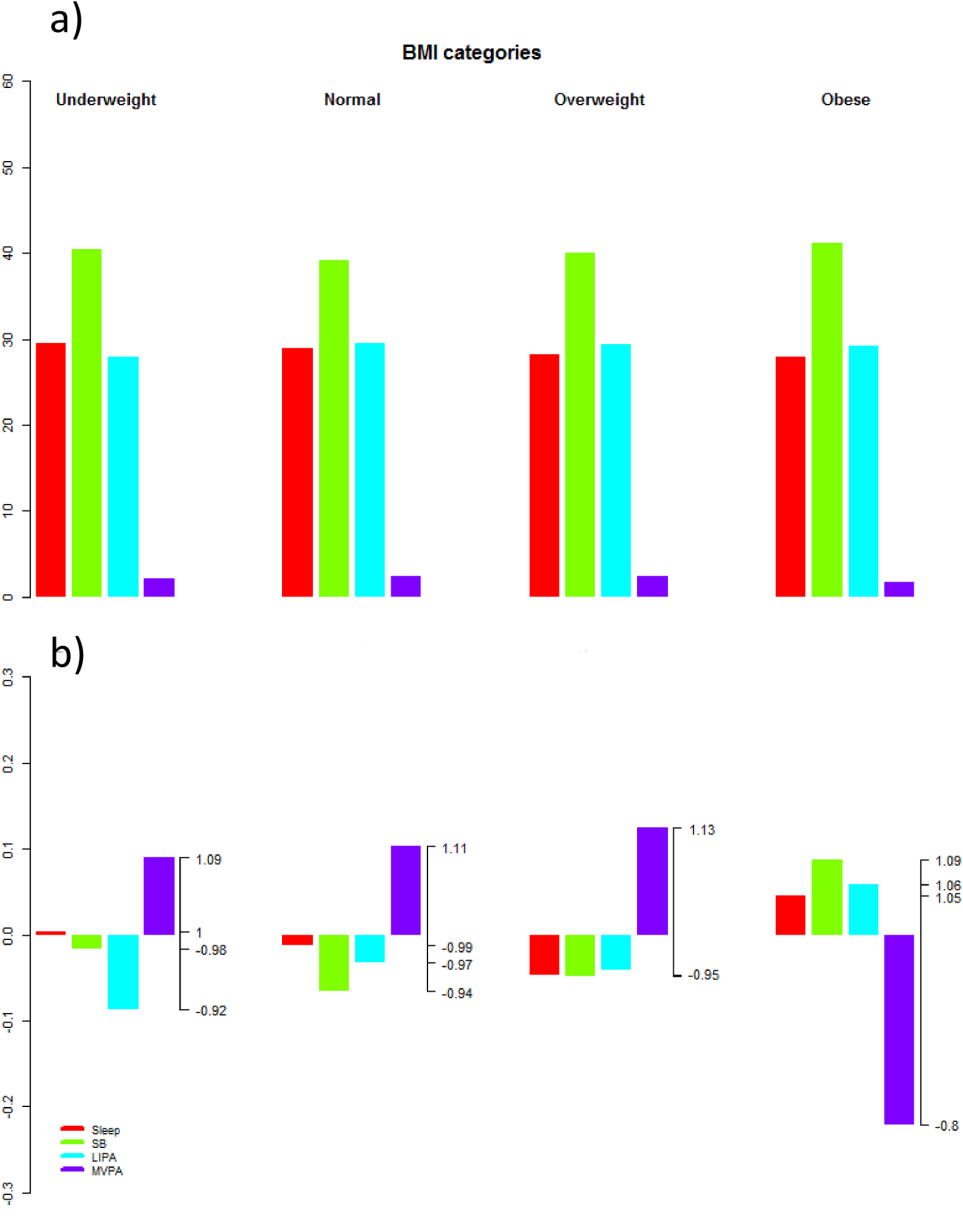
Composition of the day by BMI groups as a) proportion of time spent in sleep, SB, LIPA and MVPA and b) compositional analysis of the relative importance of the group mean time spent in sleep, SB, LIPA and MVPA with respect to the overall mean time composition. In b) the left axis gives the log-ratio value and the right axis displays the actual proportion relative to the mean composition (e.g. 1.25 means 1.25 times the compositional mean or a proportion higher by 25%).

**Fig 3:**
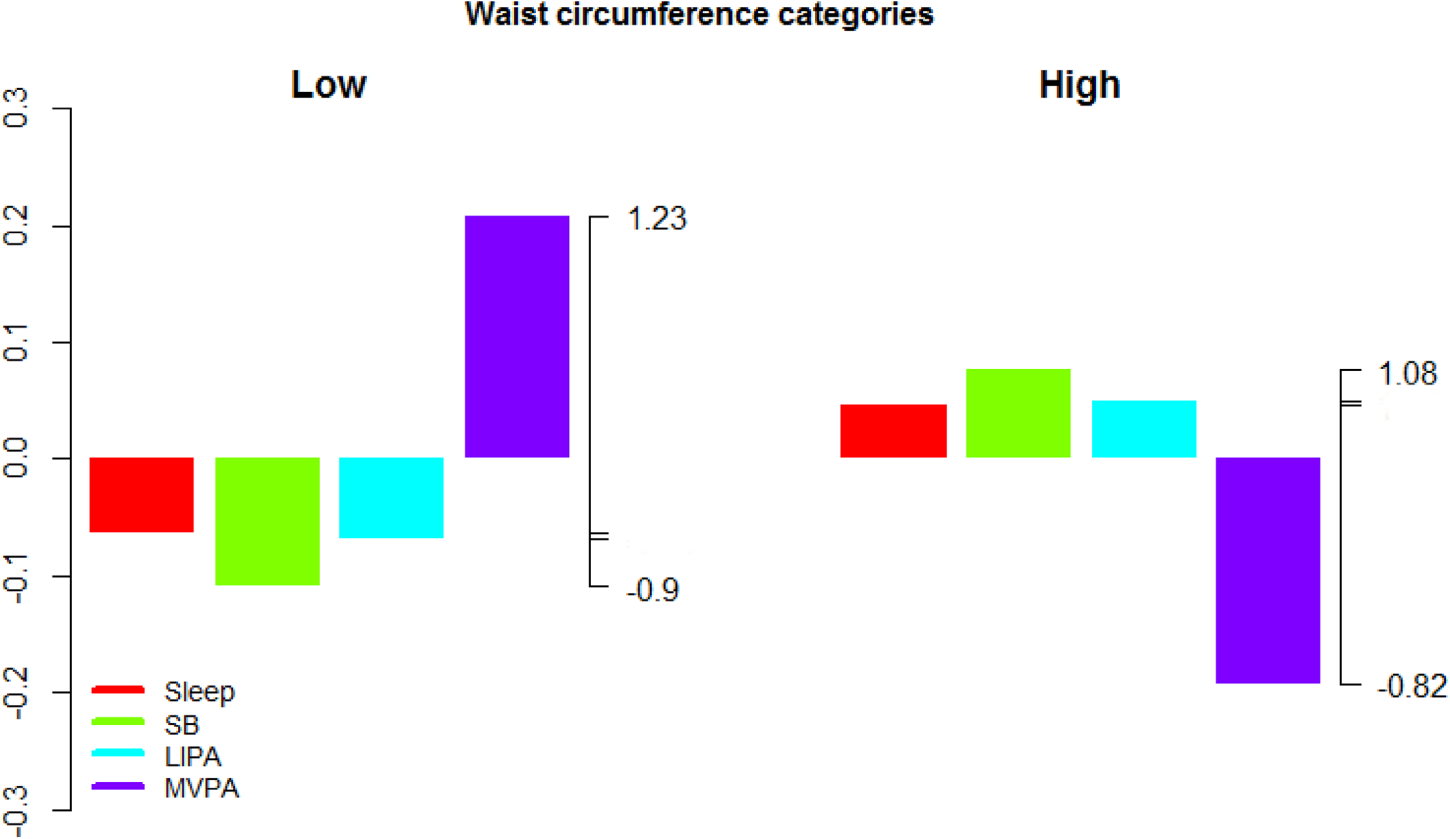
Compositional analysis of the relative importance of the group mean time spent in sleep, SB, LIPA and MVPA with respect to the overall mean time composition by group of waist circumference. The left axis gives the log-ratio value and the right axis displays the actual proportion relative to the mean composition (e.g. 1.25 means 1.25 times the compositional mean or a proportion higher by 25%). Grouping by waist circumference was carried out by gender according to the following thresholds; 102 cm for men and 88 cm for women.

**Fig 4:**
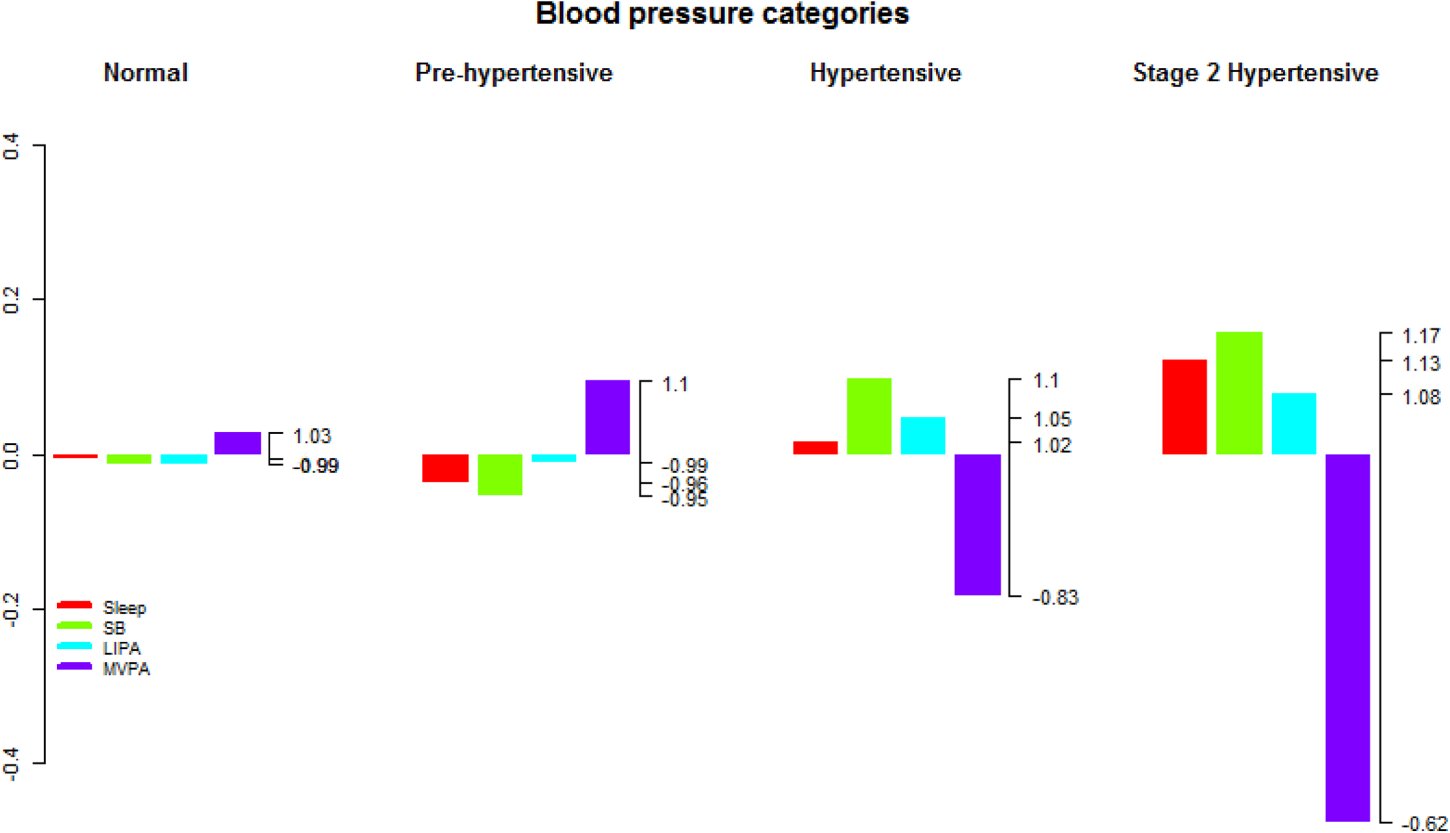
Compositional analysis of the relative importance of the group mean time spent in sleep, SB, LIPA and MVPA with respect to the overall mean time composition by group of blood pressure. The left axis gives the log-ratio value and the right axis displays the actual proportion relative to the mean composition (e.g. 1.25 means 1.25 times the compositional mean or a proportion higher by 25%). Grouping by blood pressure was according to the National Institute of Health categories.

**Fig 5:**
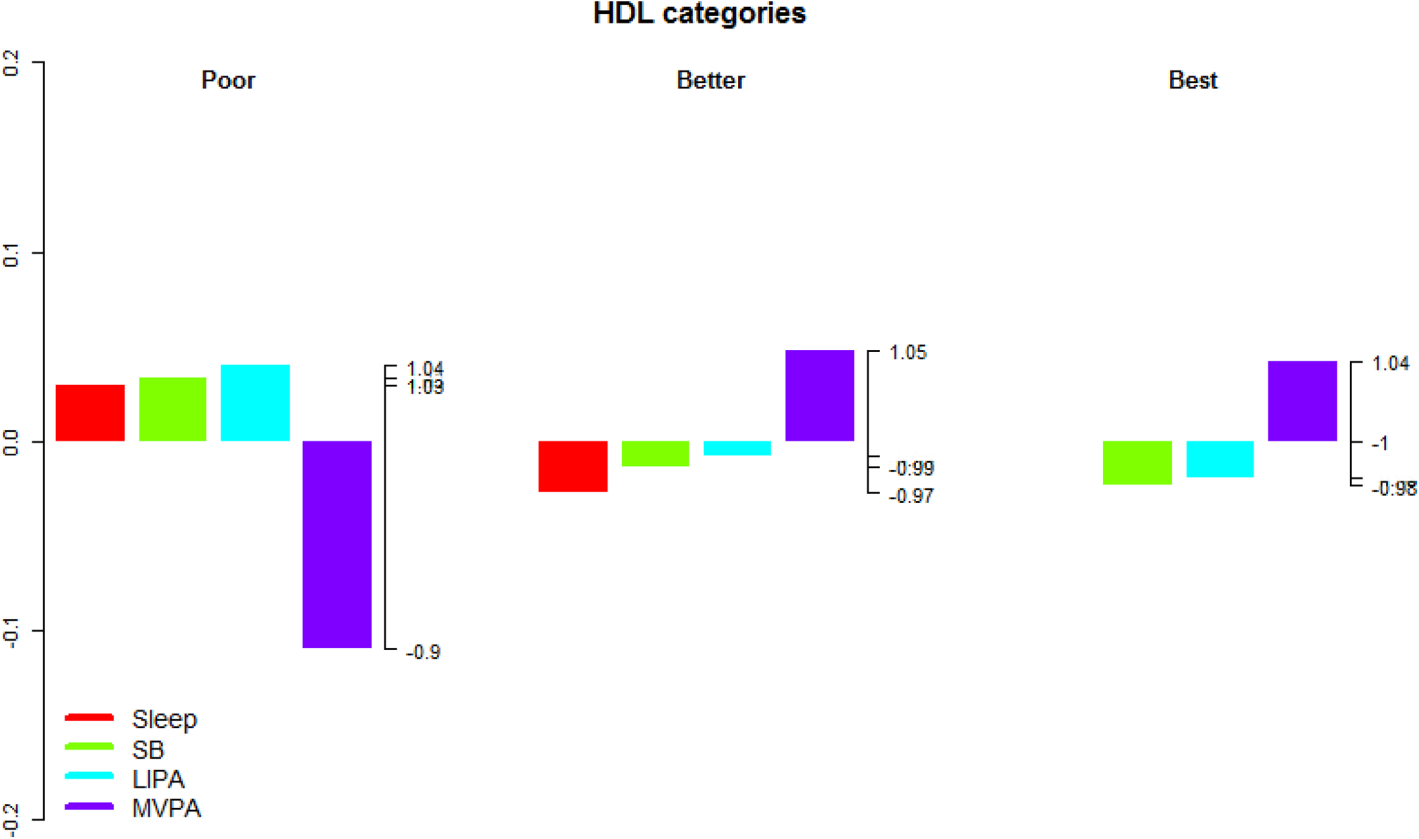
Compositional analysis of the relative importance of the group mean time spent in sleep, SB, LIPA and MVPA with respect to the overall mean time composition by group of blood HDL concentration. The left axis gives the log-ratio value and the right axis displays the actual proportion relative to the mean composition (e.g. 1.25 means 1.25 times the compositional mean or a proportion higher by 25%). Grouping by blood HDL concentration was according to the American Heart Association guidelines for treatment of cholesterol [58].

**Fig 6:**
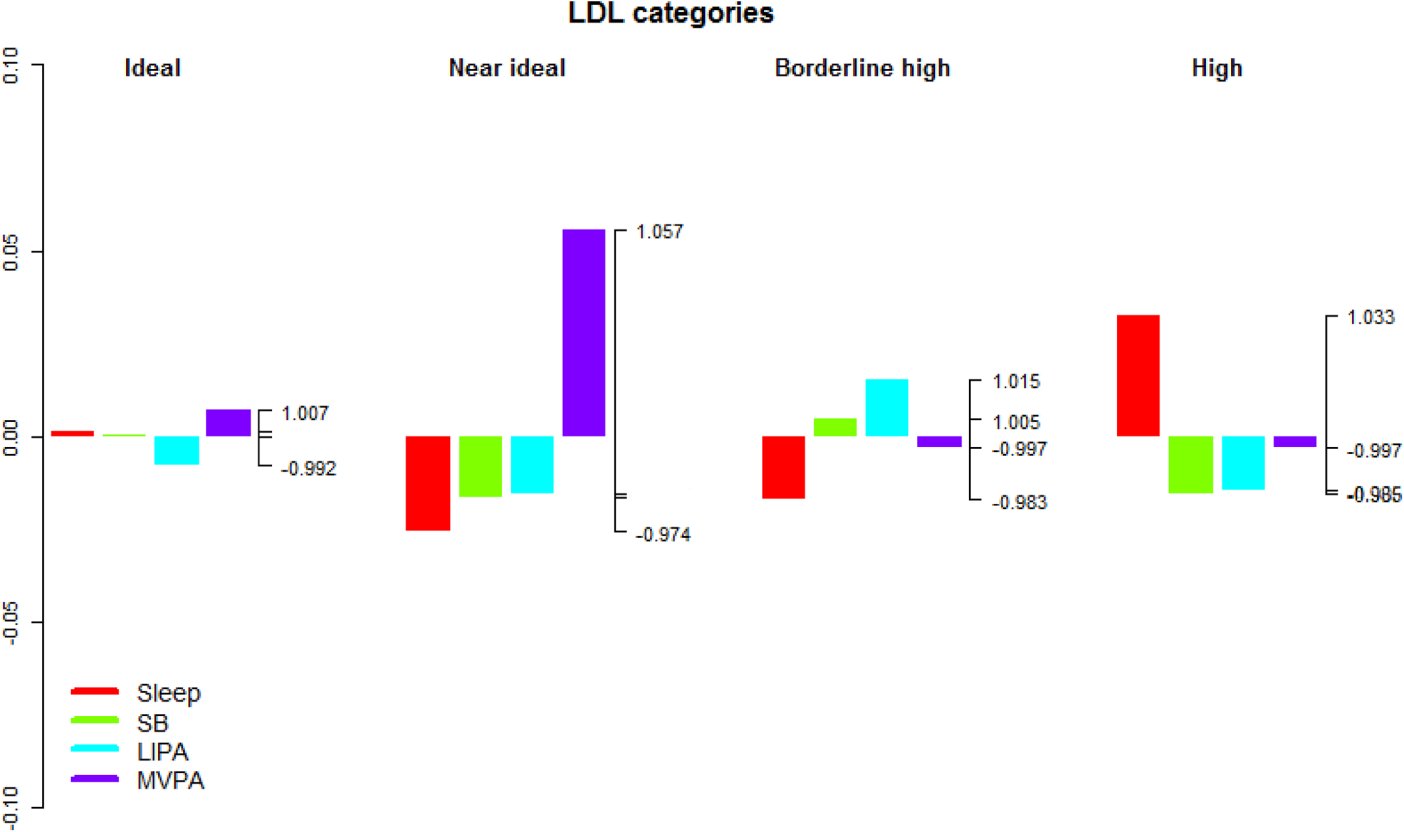
Compositional analysis of the relative importance of the group mean time spent in sleep, SB, LIPA and MVPA with respect to the overall mean time composition by group of blood LDL concentration. The left axis gives the log-ratio value and the right axis displays the actual proportion relative to the mean composition (e.g. 1.25 means 1.25 times the compositional mean or a proportion higher by 25%). Grouping by blood LDL concentration was according to the American Heart Association guidelines for treatment of cholesterol [58].

**Fig 7:**
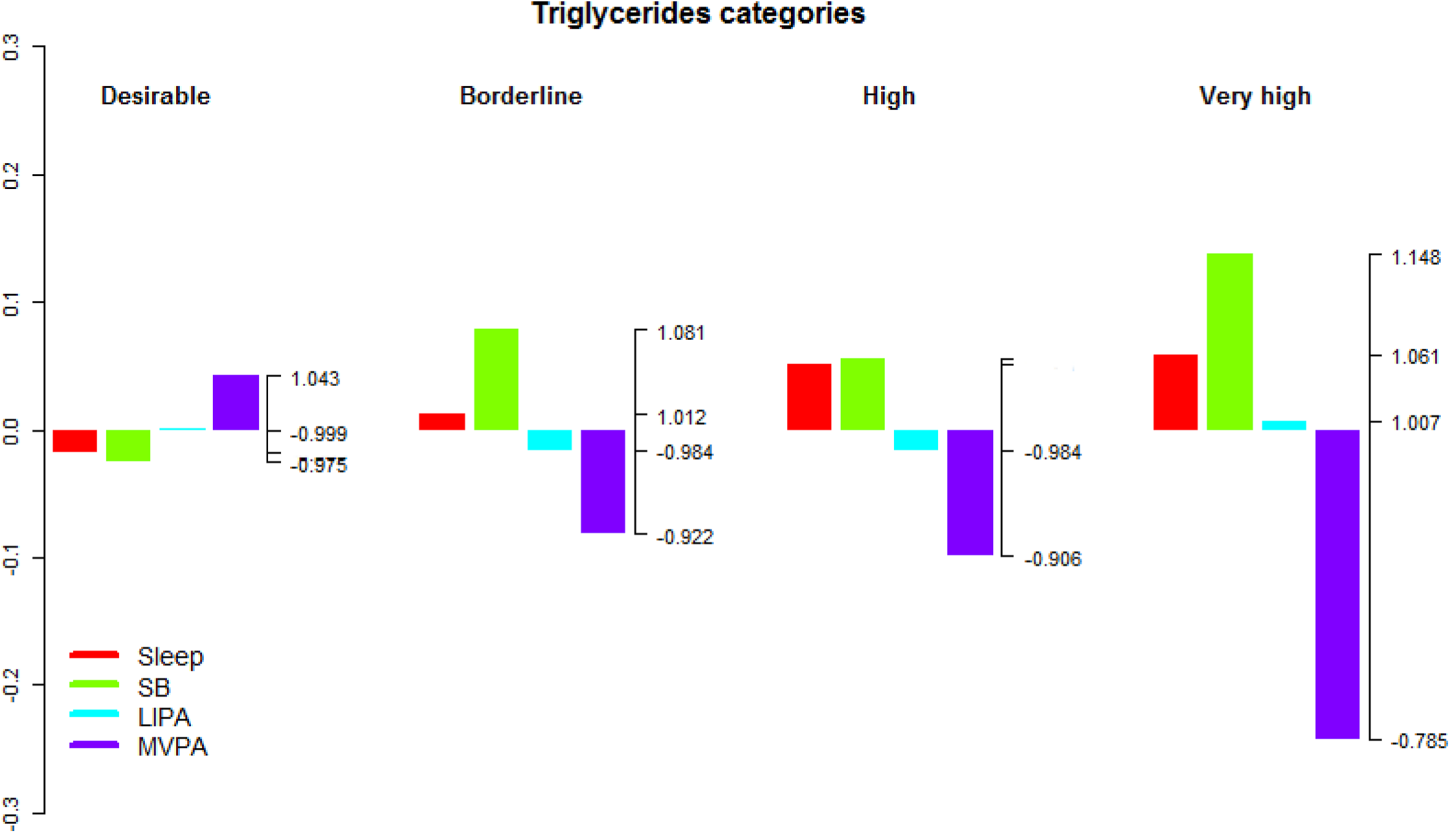
Compositional analysis of the relative importance of the group mean time spent in sleep, SB, LIPA and MVPA with respect to the overall mean time composition by group of blood triglycerids concentration. The left axis gives the log-ratio value and the right axis displays the actual proportion relative to the mean composition (e.g. 1.25 means 1.25 times the compositional mean or a proportion higher by 25%). Grouping by blood triglycerids concentration was according to the American Heart Association guidelines for treatment of cholesterol [58].

**Fig 8:**
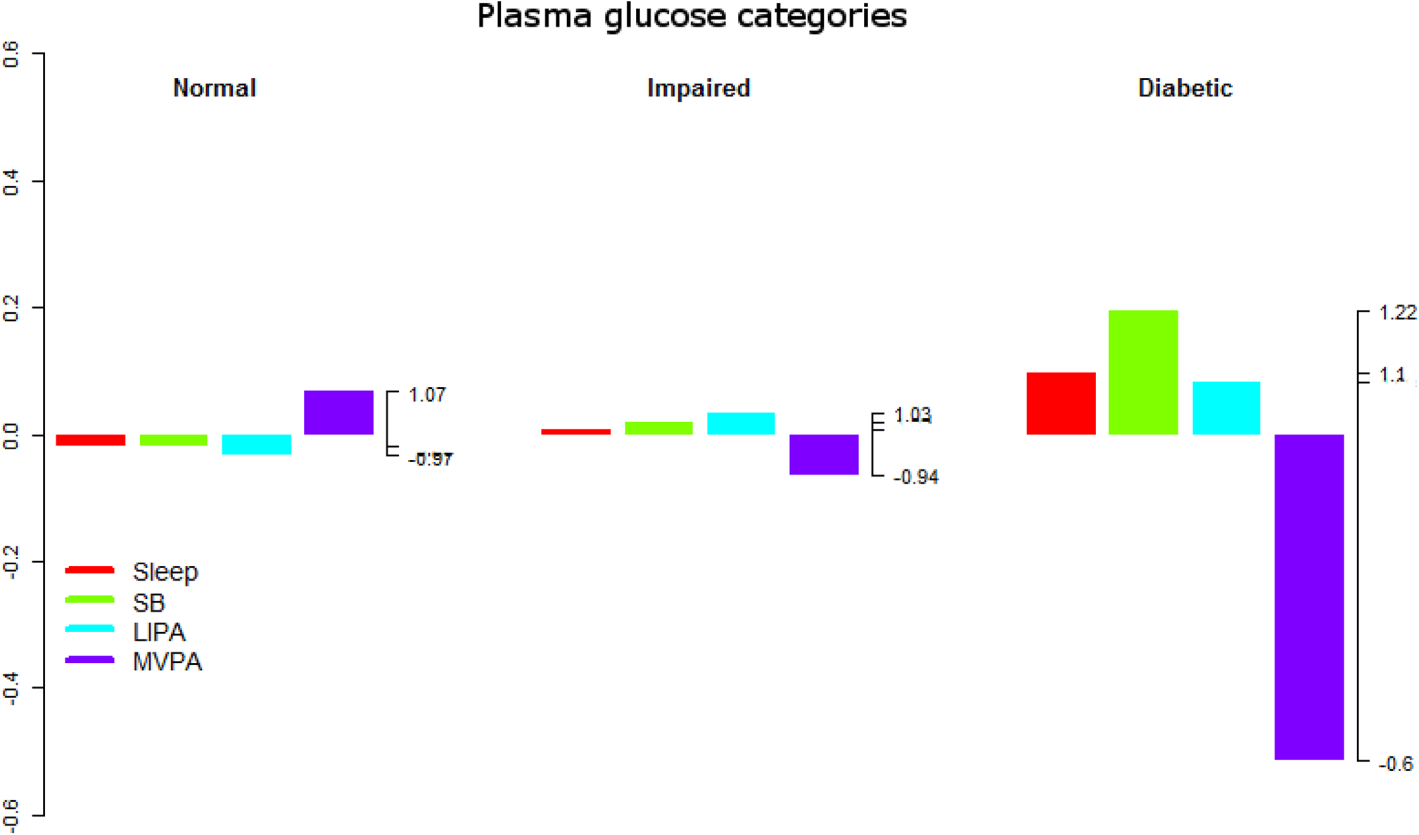
Compositional analysis of the relative importance of the group mean time spent in sleep, SB, LIPA and MVPA with respect to the overall mean time composition by group of plasma blood glucose concentration. The left axis gives the log.ratio value and the right axis displays the actual proportion relative to the mean composition (e.g. 1.25 means 1.25 times the compositional mean or a proportion higher by 25%). Grouping by plasma blood glucose concentration was according to the American Diabetes Association guidelines.

**Fig 9:**
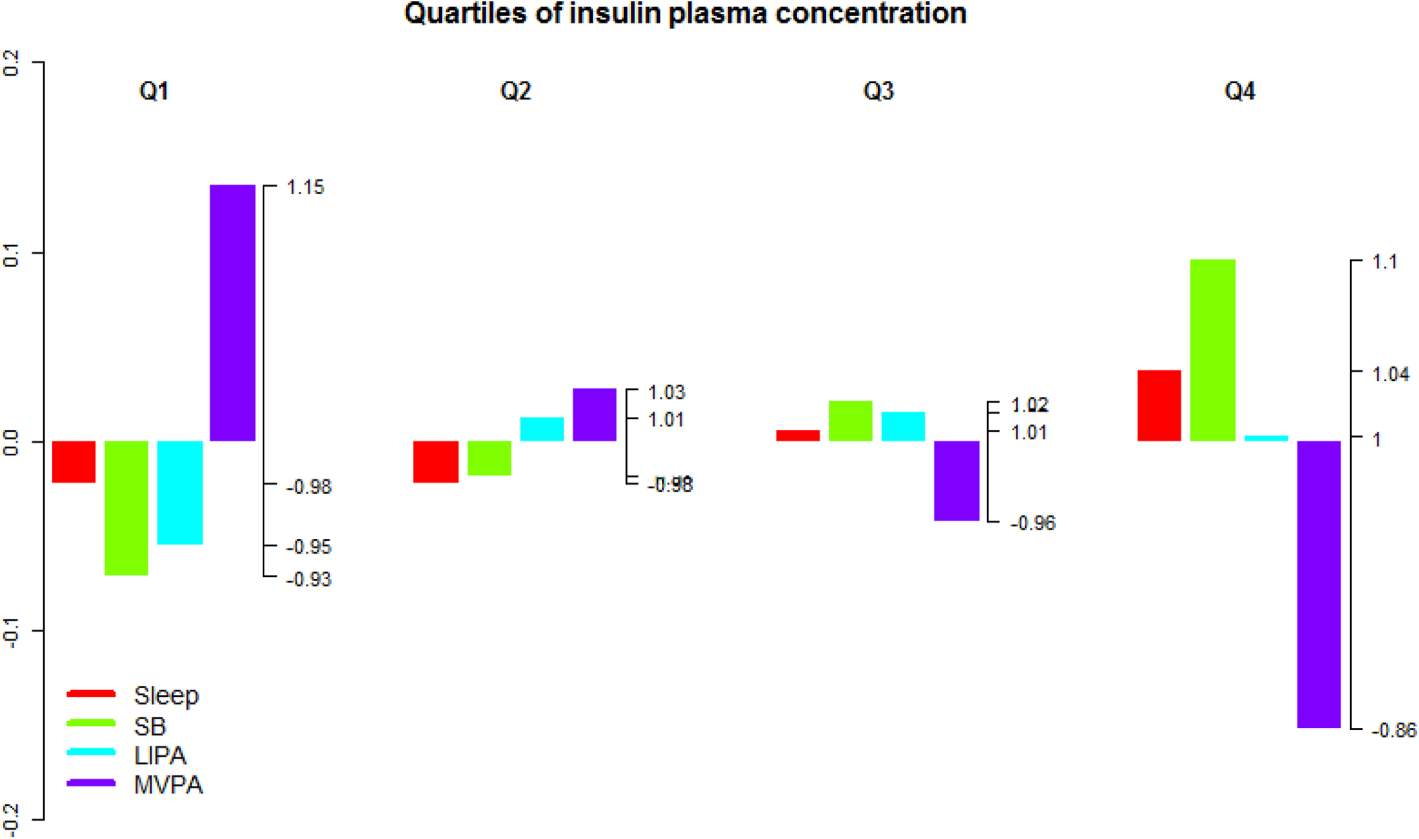
Compositional analysis of the relative importance of the group mean time spent in sleep, SB, LIPA and MVPA with respect to the overall mean time composition by group of plasma blood insulin concentration. The left axis gives the log-ratio value and the right axis displays the actual proportion relative to the mean composition (e.g. 1.25 means 1.25 times the compositional mean or a proportion higher by 25%). Grouping by plasma blood insulin concentration was based on quartiles.

**Fig 10:**
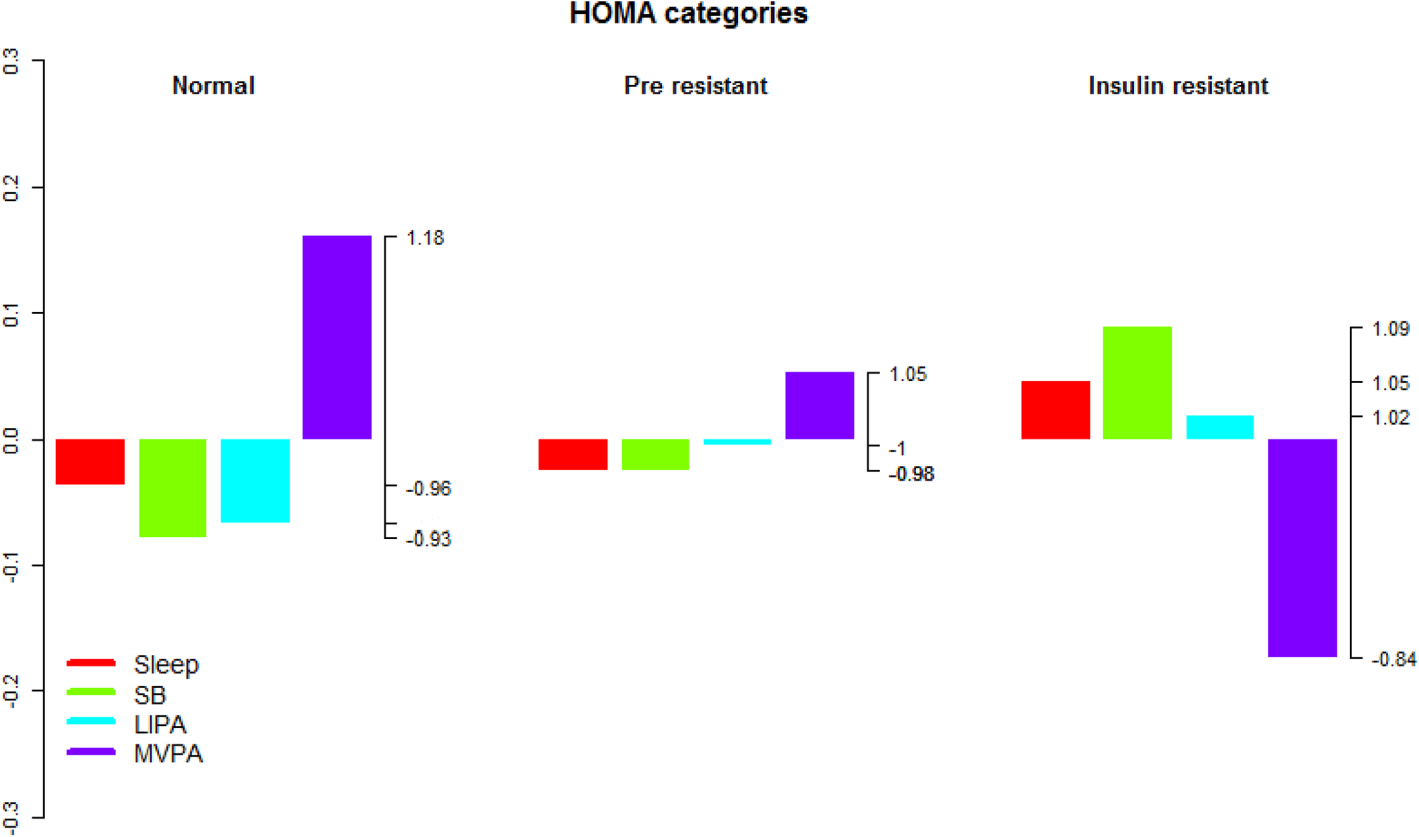
Compositional analysis of the relative importance of the group mean time spent in sleep, SB, LIPA and MVPA with respect to the overall mean time composition by group of HOMA. The left axis gives the log-ratio value and the right axis displays the actual proportion relative to the mean composition (e.g. 1.25 means 1.25 times the compositional mean or a proportion higher by 25%). Grouping by HOMA categories.

The composition of the day for participants grouped by BMI is presented in Fig 2A using standard barplots of the absolute proportions of time. As sleep, SB and LIPA values dominate, it is difficult to appreciate the relative differences between these groups. A compositional analysis alternative is presented in Fig 2B, where the relative distribution of times for each group is presented as the log-ratio between the group compositional mean and the overall compositional mean after centering the data. With this representation, it is easier to see that in the obese group the proportion of time spent in MVPA is reduced by 20% relatively to the overall mean composition and to appreciate the transfer of time between behaviors. Similar plots for clinically significant groupings or quartiles of the other outcomes are shown on Figs 3–10. The method of computation is detailed in S2 File.

### Linear regression models

Results of linear regression models are presented in Table 3. The models’ fit to the data was in line with previous reports [1,6,10,14–17], with moderate amount of variance explained for systolic blood pressure (R^2^_adj_ = 0.21), HDL (R^2^_adj_ = 0.22), CRP (R^2^_adj_ = 0.26), plasma glucose (R^2^_adj_ = 0.33), insulin (R^2^_adj_ = 0.40) and HOMA (R^2^_adj_ = 0.42). Less variance was explained by the models for BMI (R^2^_adj_ = 0.10), waist circumference (R^2^_adj_ = 0.12), diastolic blood pressure (R^2^_adj_ = 0.12), triglycerides (R^2^_adj_ = 0.12) and very little for LDL (R^2^_adj_ = 0.04). The amount of variance explained varied little between the SINGLE and CODA models. In the SINGLE models, the proportion of time spent sleeping was statistically significantly associated with higher triglycerides and CRP levels, but associated with lower BMI and diastolic blood pressure. The proportion of time spent sedentary was deleteriously associated with BMI, waist circumference, HDL, triglycerides, plasma insulin and HOMA. Both proportion of time spent in LIPA and MVPA were associated with better outcomes for waist circumference, triglycerides, plasma insulin and HOMA, with MVPA showing much higher effect sizes. MVPA was also beneficially associated with BMI and HDL. For all statistically significant associations the effect sizes of SB and LIPA were comparable but in opposite directions.

**Table 3:** Single (SINGLE) and compositional (CODA) behaviour models for cardiometabolic markers for proportion of the day spent in each behaviour; sleep, sedentary behaviors (SB), light activity (LIPA) and moderate to vigorous activity (MVPA). Statistically significant associations at the 95% confidence level (p < 0.05) are highlighted in bold. Note that the 95% confidence intervals (CI) for the regression γ in the CODA models are omitted as they are meaningless in a compositional paradigm. The models were adjusted for age, gender, ethnicity/race, self-reported health, diagnosis of health conditions, educational level, social economic status, smoking status, alcohol consumption, total daily average dietary calorie intake, fat intake, caffeine intake, usage of medications for high blood pressure and/or diabetes, by backward elimination (with predictor retained if p < 0.2). This adjustment is the same for the SINGLE and CODA models for a given outcome (marker). The regression coefficient for the CODA model corresponds to change in the log-ratio of the given behavior to the others.

SINGLE									
Marker	Sleep		SB			LIPA		MVPA	
	B [95%ci]	p	B [95%CI]	p		B [95%CI]	p	B [95%CI]	p
BMI	**-8.46 [-13.56–3.37]**	**0.001**	**6.85 [3.68 10.03]**	**<0.001**		-2.21 [-5.75 1.34]	0.222	**-42.29 [-57.80–26.79]**	**<0.001**
Waist circumference	-5.61 [-17.71 6.48]	0.363	**17.10 [9.59 24.61]**	**<0.001**		**-11.39 [-19.75–3.03]**	**0.008**	**-134.13 [-170.4–97.8]**	**<0.001**
Systolic Blood Pressure	-9.07 [-22.98 4.85]	0.201	-1.32 [-10.66 8.02]	0.781		5.39 [-4.99 15.77]	0.308	21.40 [-21.00 63.81]	0.322
Diastolic Blood Pressure	**-11.31 [-21.84–0.79]**	**0.035**	1.54 [-5.54 8.60]	0.6707		4.26 [-3.59 12.12]	0.287	2.21 [-29.91 34.32]	0.892
HDL^†^	0.09 [-0.12 0.31]	0.388	**-0.17 [-0.31–0.03]**	**0.018**		0.08 [-0.08 0.24]	0.314	**1.39 [0.73 2.04]**	**<0.001**
LDL	0.86 [-0.25 1.97]	0.128	0.05 [-0.70 0.81]	0.888		-0.53 [-1.37 0.31]	0.215	-0.47 [-4.05 3.12]	0.797
Triglycerides^†^	**1.02 [0.34 1.69]**	**0.003**	**0.74 [0.29 1.19]**	**0.001**		**-1.26 [-1.76–0.76]**	**<0.001**	**-4.56 [-6.69–2.44]**	**<0.001**
CRP^†^	**1.29 [0.33 2.24]**	**0.009**	0.33 [-0.31 0.96]	0.312		-0.73 [-1.43–0.03]	0.041	**-0.73 [-1.43–0.03]**	**0.041**
Plasma Glucose^†^	0.20 [-0.01 0.41]	0.053	-0.09 [-0.23 0.05]	0.194		-0.01 [-0.16 0.15]	0.986	-0.01 [-0.18 0.15]	0.986
Plasma Insulin^†^	0.22 [-0.43 0.87]	0.514	**0.63 [0.20 1.07]**	**0.003**		**-0.72 [-1.19–0.25]**	**0.003**	**-3.02 [-5.14–0.87]**	**0.006**
HOMA^†^	0.51 [-0.19 1.21]	0.156	**0.62 [0.14 1.09]**	**0.011**		**-0.87 [-1.40–0.34]**	**0.001**	**-3.24 [-5.54–0.94]**	**0.006**
CODA									
Marker	Sleep		SB			LIPA	MVPA		Composition
	γ_1_^1^	p	γ_1_^2^	p	γ_1_^3^	p	γ_1_^4^	P	p
BMI	**-1.40**	**0.009**	**1.40**	**0.002**	**0.98**	**0.029**	**-0.98**	**<0.001**	**<0.001**
Waist circumference	-0.41	0.749	**2.41**	**0.023**	0.96	0.368	**-2.96**	**<0.001**	**<0.001**
Systolic Blood Pressure	-1.23	0.404	-0.04	0.972	1.30	0.301	-0.02	0.966	**<0.001**
Diastolic Blood Pressure	**-2.22**	**0.048**	1.03	0.269	0.70	0.461	0.49	0.198	**0.003**
HDL^†^	0.02	0.286	-0.03	0.096	-0.01	0.549	**0.02**	**0.012**	0.121
LDL	0.18	0.131	-0.04	0.664	-0.10	0.323	-0.03	0.410	0.434
Triglycerides^†^	**0.17**	**0.018**	0.09	0.132	**-0.21**	**<0.001**	-0.04	0.062	**<0.001**
CRP^†^	**0.23**	**0.024**	-0.04	0.562	-0.06	0.472	**-0.12**	**<0.001**	**<0.001**
Plasma Glucose^†^	**0.04**	**0.044**	-0.03	0.070	-0.01	0.691	-0.01	0.811	**<0.001**
Plasma Insulin^†^	0.05	0.488	0.10	0.077	**-0.13**	**0.033**	-0.02	0.316	**<0.001**
HOMA^†^	0.10	0.158	0.07	0.249	**-0.15**	**0.020**	-0.03	0.295	**<0.001**

^†^Log transformed outcome.

The CODA models show that the relative distribution of time amongst the four behaviors as a whole is statistically significantly associated with all outcomes (BMI p<0.001, waist circumference p<0.001, systolic blood pressure p<0.001, diastolic blood pressure p = 0.003, triglycerides p<0.001, plasma glucose p<0.001, plasma insulin p<0.001, HOMA p<0.001) except cholesterol levels (HDL p = 0.121 and LDL p = 0.434). For BMI, the proportion of time spent in each behavior compared to the other three was statistically significantly detrimentally associated with SB (B = 1.40, p = 0.002) and LIPA (B = 0.98 and p = 0.029) but favorably with sleep (B = -1.40, p = 0.009) and MVPA (B = -0.098, p<0.001). Interestingly, the same effect sizes but with opposite effects were observed for SB and sleep. However, for waist circumference only SB (B = 2.41, p = 0.023) and MVPA (B = -2.96, p<0.001) showed statistically significant associations with opposite effect and similar strength. No parts of the composition were significantly associated with systolic blood pressure, but sleep was favorably associated with diastolic blood pressure (B = -2.22, p = 0.048). The proportion of time spent in MVPA was associated beneficially with HDL levels (B = 0.02, p = 0.012) and this was the only part of the composition statistically significantly associated with this outcome. Statistical evidence of association between MVPA and lower CRP levels was also obtained (B = -0.12, p<0.001). Sleep time was statistically significantly associated with higher CRP (B = 0.23, p = 0.024).

Statistical support was obtained to conclude that the contrast between LIPA and the other behaviors was favorably associated with triglycerides (B = -0.21, p<0.001), plasma insulin (B = -0.13, p = 0.033), and HOMA (B = -0.15,p = 0.020); while SB was associated with worse outcomes only for obesity markers; and sleep deleteriously associated with triglycerides (B = 0.17, p = 0.018), CRP (B = 0.23, p = 0.024) and plasma glucose (B = 0.04, p = 0.044).

### Effect of time re-allocation

In order to further understand the role played by the proportion of time spent in each behavior of the composition of the day, we estimated from the CODA models the effect on the outcome of transferring 10 minutes from one behavior to another one around the average composition (Table 1). The absolute values of these estimates will vary if the time re-allocation is computed around a different starting composition, but the direction and relative size of the effects should be the same. We chose 10 minutes as this is the smallest unit of change in activity recognized to have beneficial health effect [2,3]. The results are presented in Table 4 and the computation is described in detail in S2 File.

**Table 4:** Change matrices, showing the effect on each outcome of taking away 10 minutes of time from the behavior in columns and allocating them to the behavior in rows. The effect is computed for time re-allocation around the average composition (Table 1) and expressed as % change in the outcome about their mean value in the sample (BMI = 28.9 kg/m^2^, waist circumference = 97.7 cm, systolic blood pressure = 120.1 mmHg, diastolic blood pressure = 70.8 mmHg, LDL = 3.06 mmol/L, HDL = 1.41 mmol/L, triglycerides = 1.65 mmol/L, CRP = 0.44 mg/dL, glucose = 5.74 mmol/L, insulin = 69.12 pmol/L, HOMA = 3.04). The behaviours are: sleep, sedentary behaviour (SB), light activity (LIPA) and moderate to vigorous activity (MVPA).

BMI					WaistCircumference					SystolicBloodPressure				
	Sleep	SB	LIPA	MVPA		Sleep	SB	LIPA	MVPA		Sleep	SB	LIPA	MVPA
Sleep		-0.002	-0.002	-0.070	Sleep		-0.001	-0.001	0.290	Sleep		0.000	-0.001	-0.090
SB	0.003		0.000	1.210	SB	0.001		0.001	0.840	SB	0.000		-0.001	-0.001
LIPA	0.003	0.000		0.850	LIPA	0.001	0.000		0.550	LIPA	0.001	0.000		0.120
MVPA	0.000	-0.001	-0.001		MVPA	-0.001	-0.001	-0.001		MVPA	0.000	0.000	0.000	
DiastolicBloodPressure					LDL					Log(HDL)				
	Sleep	SB	LIPA	MVPA		Sleep	SB	LIPA	MVPA		Sleep	SB	LIPA	MVPA
Sleep		0.000	-0.001	-0.360	Sleep		0.001	0.002	0.640	Sleep		0.003	0.004	0.090
SB	0.001		0.000	0.070	SB	-0.002		0.001	0.000	SB	-0.006		-0.001	-2.490
LIPA	0.001	0.000		-0.004	LIPA	-0.002	0.000		-0.190	LIPA	-0.004	0.001		-1.280
MVPA	0.001	0.000	0.000		MVPA	-0.001	-0.001	0.000		MVPA	0.000	0.002	0.002	
Log (triglycerides)					Log(CRP)					Log (Glucose)				
	Sleep	SB	LIPA	MVPA		Sleep	SB	LIPA	MVPA		Sleep	SB	LIPA	MVPA
Sleep		0.002	0.031	6.370	Sleep		-0.002	-0.004	-2.100	Sleep		0.001	0.000	0.240
SB	-0.004		0.032	6.800	SB	0.005		0.000	-0.950	SB	-0.001		-0.001	-0.260
LIPA	-0.032	-0.017		-5.200	LIPA	0.004	0.000		-0.790	LIPA	-0.001	0.000		-0.030
MVPA	-0.010	-0.004	0.007		MVPA	0.003	0.001	0.001		MVPA	0.000	0.000	0.000	
Log (insulin)					Log(HOMA)									
	Sleep	SB	LIPA	MVPA		Sleep	SB	LIPA	MVPA					
Sleep		0.000	0.001	0.160	Sleep		0.000	0.008	1.530					
SB	0.001		0.002	0.490	SB	0.000		0.010	2.100					
LIPA	-0.001	-0.001		-0.190	LIPA	-0.001	-0.001		-1.490					
MVPA	0.000	0.000	0.000		MVPA	-0.002	-0.002	0.002						

The effect of re-allocating time from one behavior to another one whilst the other two are kept stable was found to be small and not symmetric. For example, for BMI the largest effect was found when 10 minutes of MVPA were displaced by 10 minutes of SB, this changed BMI by 1.21%. However, the opposite, replacing 10 minutes of SB by 10 minutes of MVPA, only changed BMI by -0.001%.

Sleep had a small positive effect on obesity markers. Small positive effects were also observed on systolic and diastolic blood pressure when displacing LIPA and MVPA with sleep, with larger effects observed when sleep replaces MVPA. Replacing MVPA with sleep changed positively log(HDL) by 0.09%, but only 0.004% and 0.003% changes were observed when sleep replaces LIPA and SB respectively. A similar, but more pronounced, pattern was observed for CRP, with sleep reducing the log(CRP) by 2.1% when replacing MVPA, but only by 0.004% and 0.002% when displacing LIPA and SB in that order. Finally, sleep showed detrimental effects on LDL, triglycerides, glucose and insulin level and HOMA, which were much stronger when sleep displaced MVPA.

SB was found to lead to higher obesity markers if it displaced any of the other behaviors, but only by sizeable amounts if it replaced MVPA. Likewise, SB only had noticeable detrimental effects when displacing MVPA for HDL, triglycerides, glucose, insulin, and HOMA. Much smaller detrimental effects were observed when SB replaced LIPA or sleep time.

Detrimental effects were observed when replacing MVPA and sleep with LIPA for both obesity markers, with stronger magnitude when LIPA replaced MVPA. On the contrary, LIPA had favorable effects on LDL, triglycerides, glucose, insulin and HOMA, substantially more pronounced when replacing sleep than when replacing SB.

Re-allocating time to MVPA from the other behaviors only had very small effect, most noticeable on lipids.

### Mapping the effect of behavior compositions on cardiometabolic outcomes

**Fig 11:**
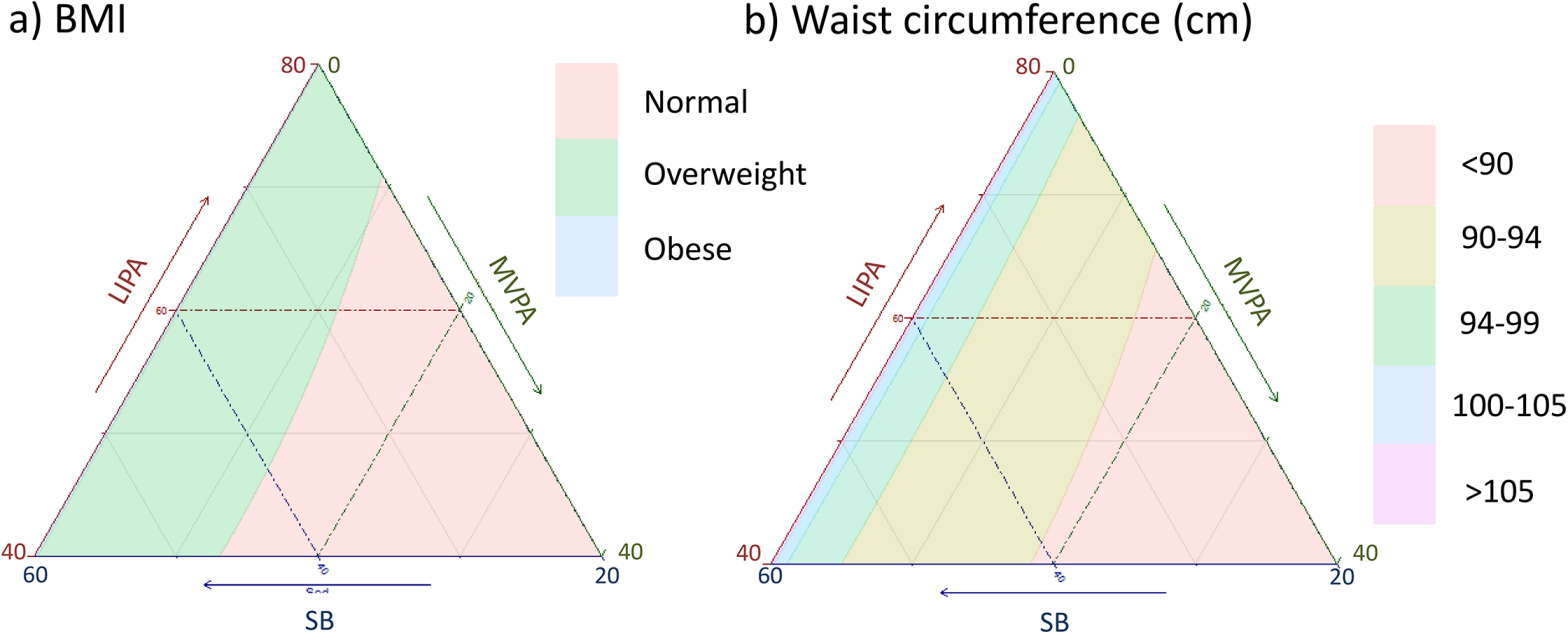
Estimated a) BMI and b) waist circumference as a function of composition of the waking day (16 hours) in proportion of time spent in sedentary behaviors (SB), Light (LIPA) and moderate to vigorous activity (MVPA) and 8 hours spent sleeping.

**Fig 12:**
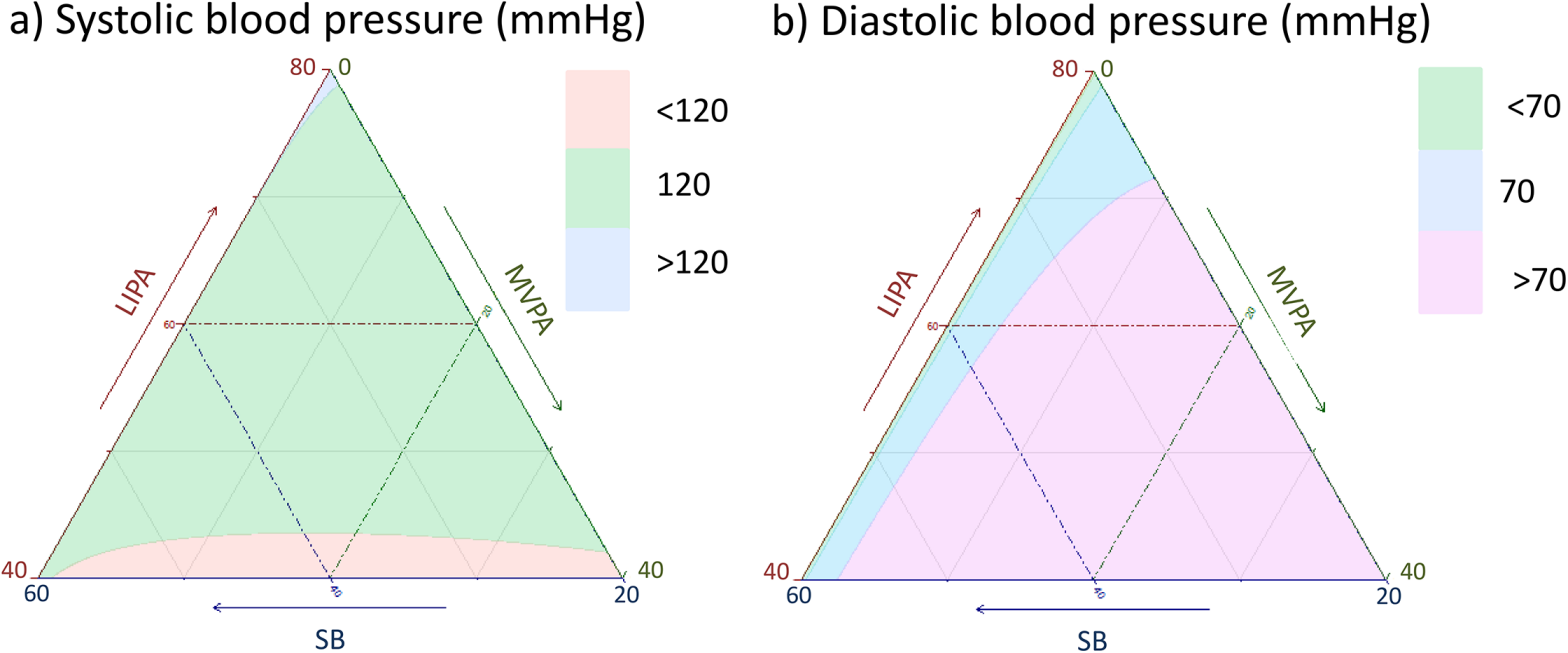
Estimated a) systolic and b) diastolic blood pressure as a function of composition of the waking day (16 hours) in proportion of time spent in sedentary behaviors (SB), Light (LIPA) and moderate to vigorous activity (MVPA) and 8 hours spent sleeping.

**Fig 13:**
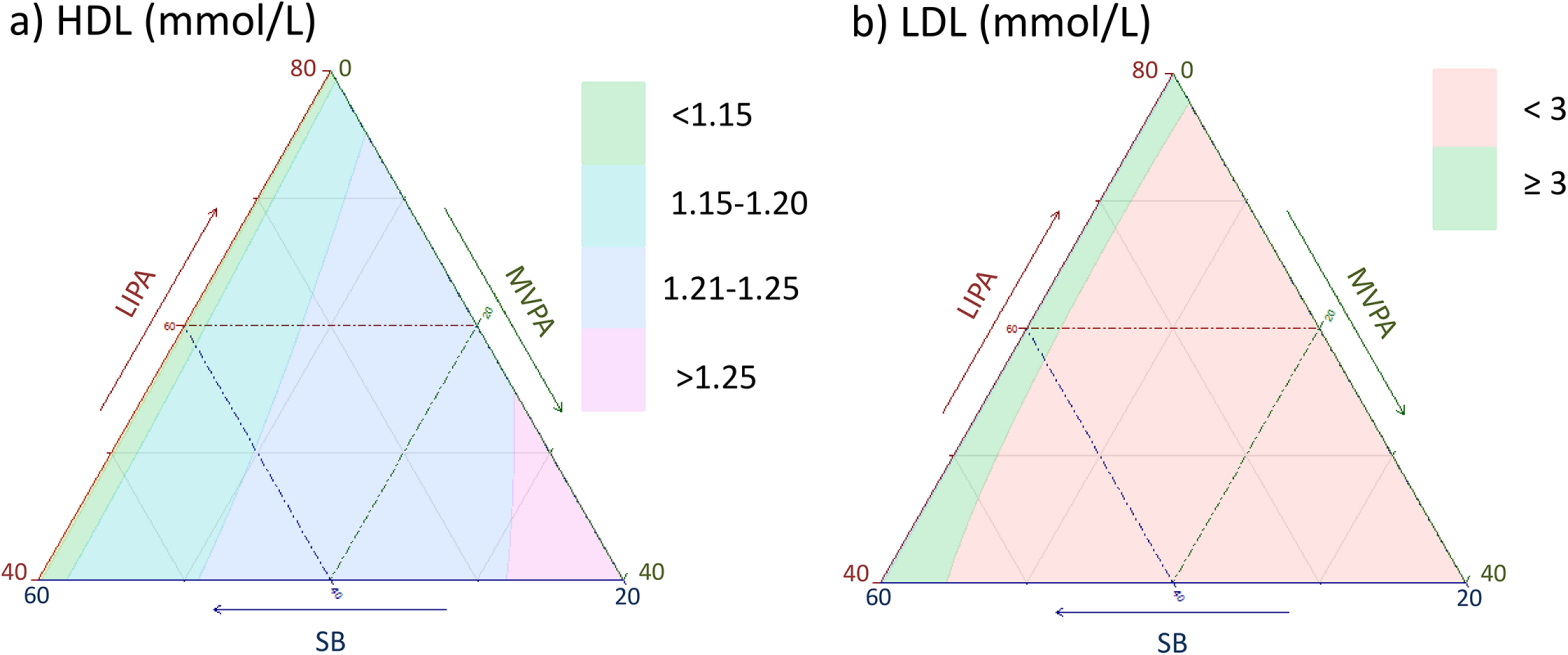
Estimated blood a) HDL and b) LDL cholesterol as a function of composition of the waking day (16 hours) in proportion of time spent in sedentary behaviors (SB), Light (LIPA) and moderate to vigorous activity (MVPA) and 8 hours spent sleeping.

**Fig 14:**
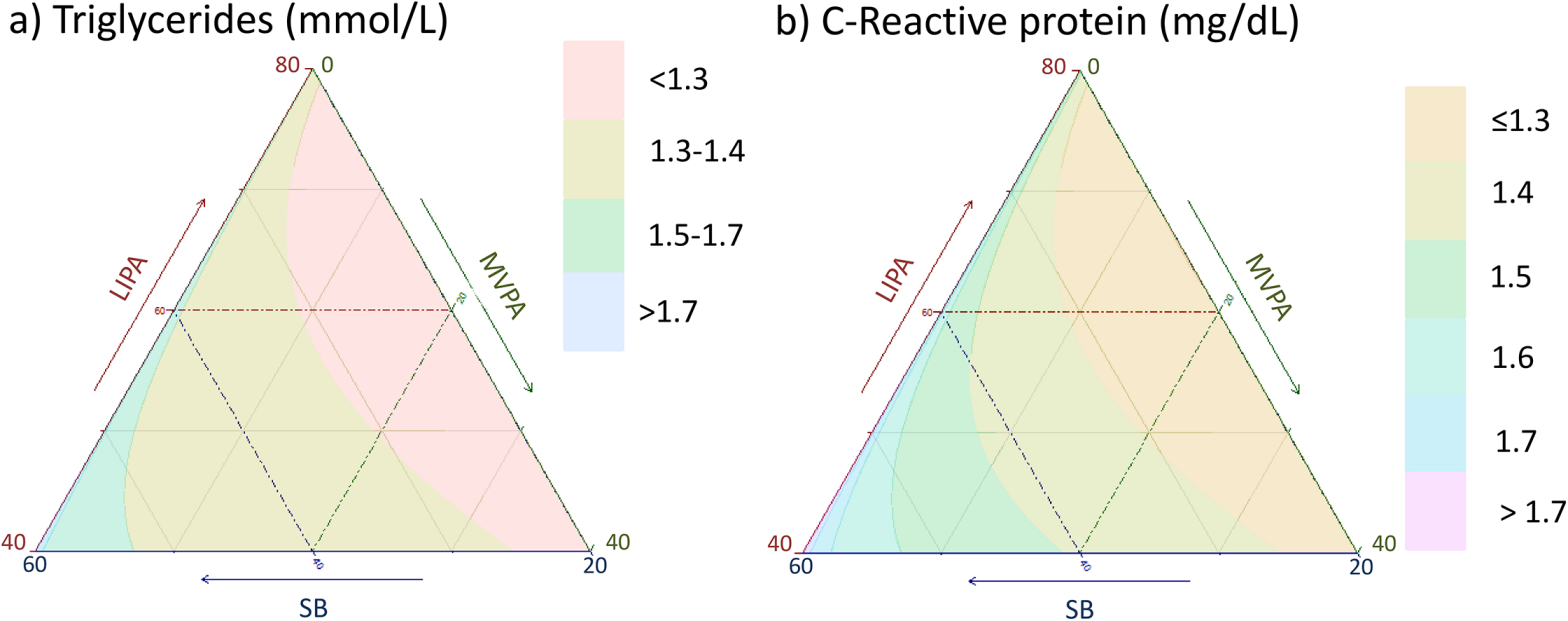
Estimated blood a) Triglycerides and b) C-reactive proteins as a function of composition of the waking day (16 hours) in proportion of time spent in sedentary behaviors (SB), Light (LIPA) and moderate to vigorous activity (MVPA) and 8 hours spent sleeping.

**Fig 15:**
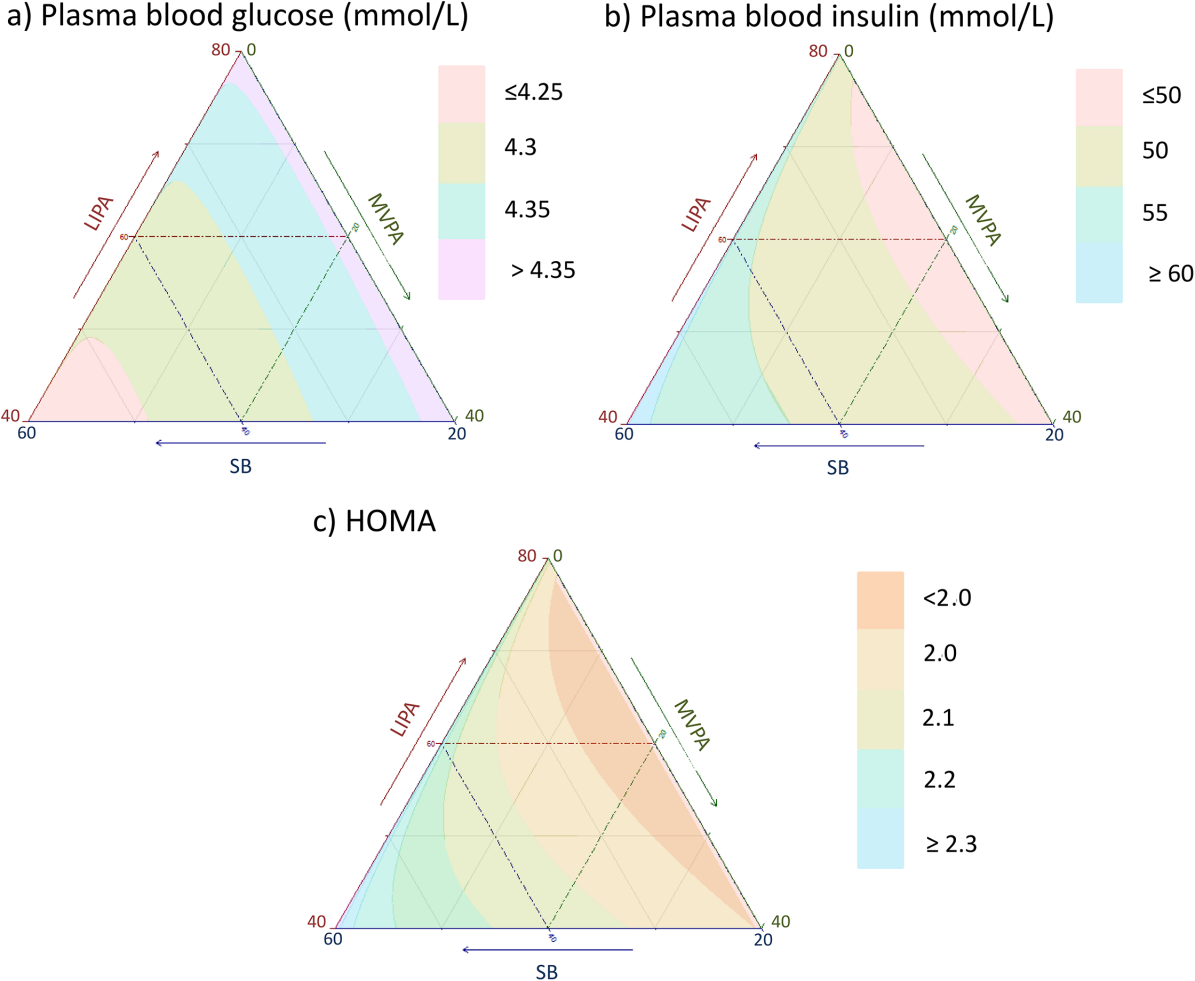
Estimated fasting plasma blood a) glucose and b) insulin and c) HOMA as a function of composition of the waking day (16 hours) in proportion of time spent in sedentary behaviors (SB), Light (LIPA) and moderate to vigorous activity (MVPA) and 8 hours spent sleeping.

We used the CODA models to estimate the effect of different compositions of the day on cardiometabolic outcomes. These are presented for each outcome as a heat map on a ternary plot showing the outcome for the composition of time spent in SB, LIPA and MVPA during the waking day (16 hours) with 8 hours of sleep time (Figs 11–15).

**Fig 16:**
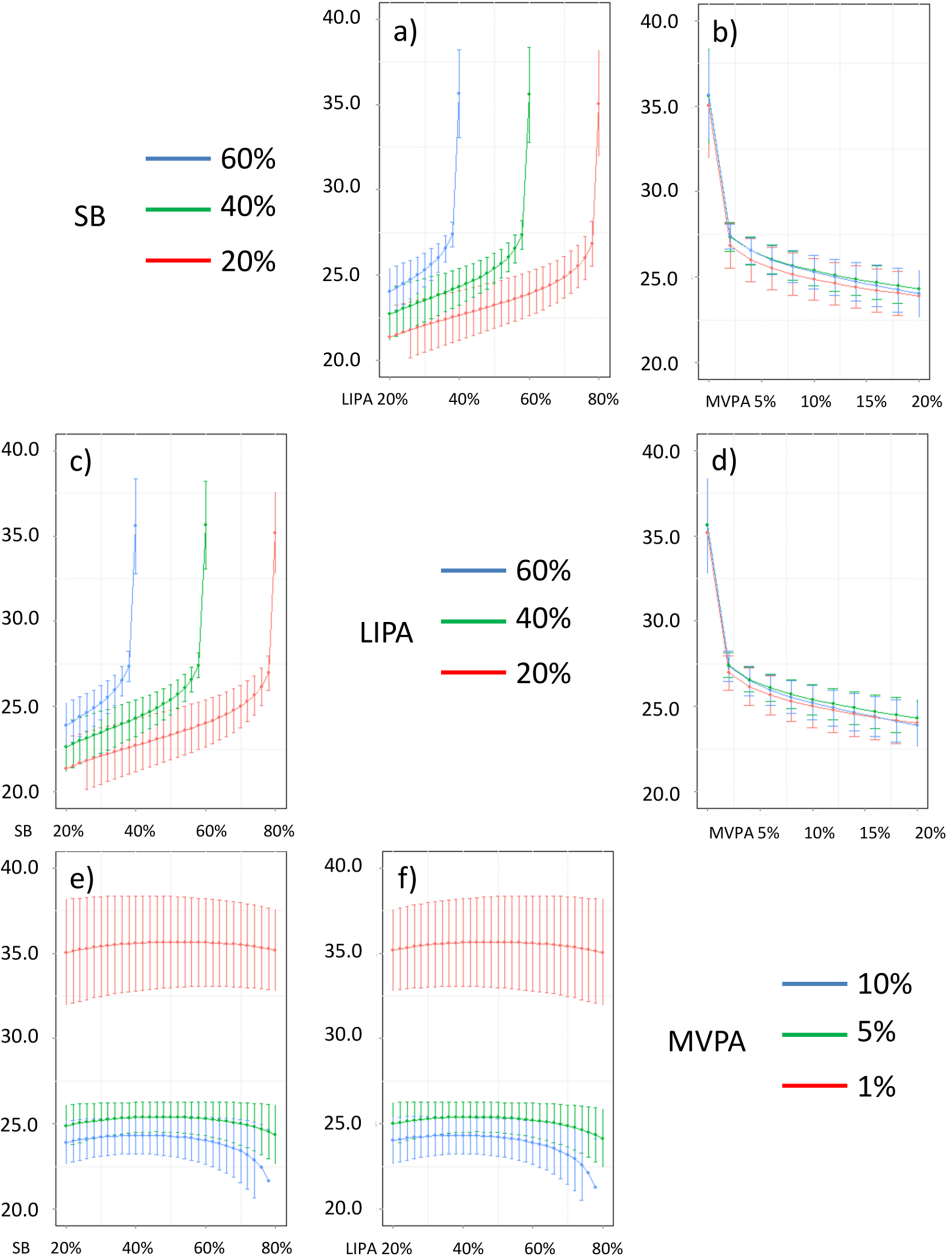
Estimated BMI (in kg/m2) for different composition as a function the proportion of a) LIPA and b) MVPA when SB is held constant, c) SB and d) MVPA when LIPA is held constant, e) SB and f)LIPA when MVPA is held constant. The plots correspond to slices of Fig 11A.

**Fig 17:**
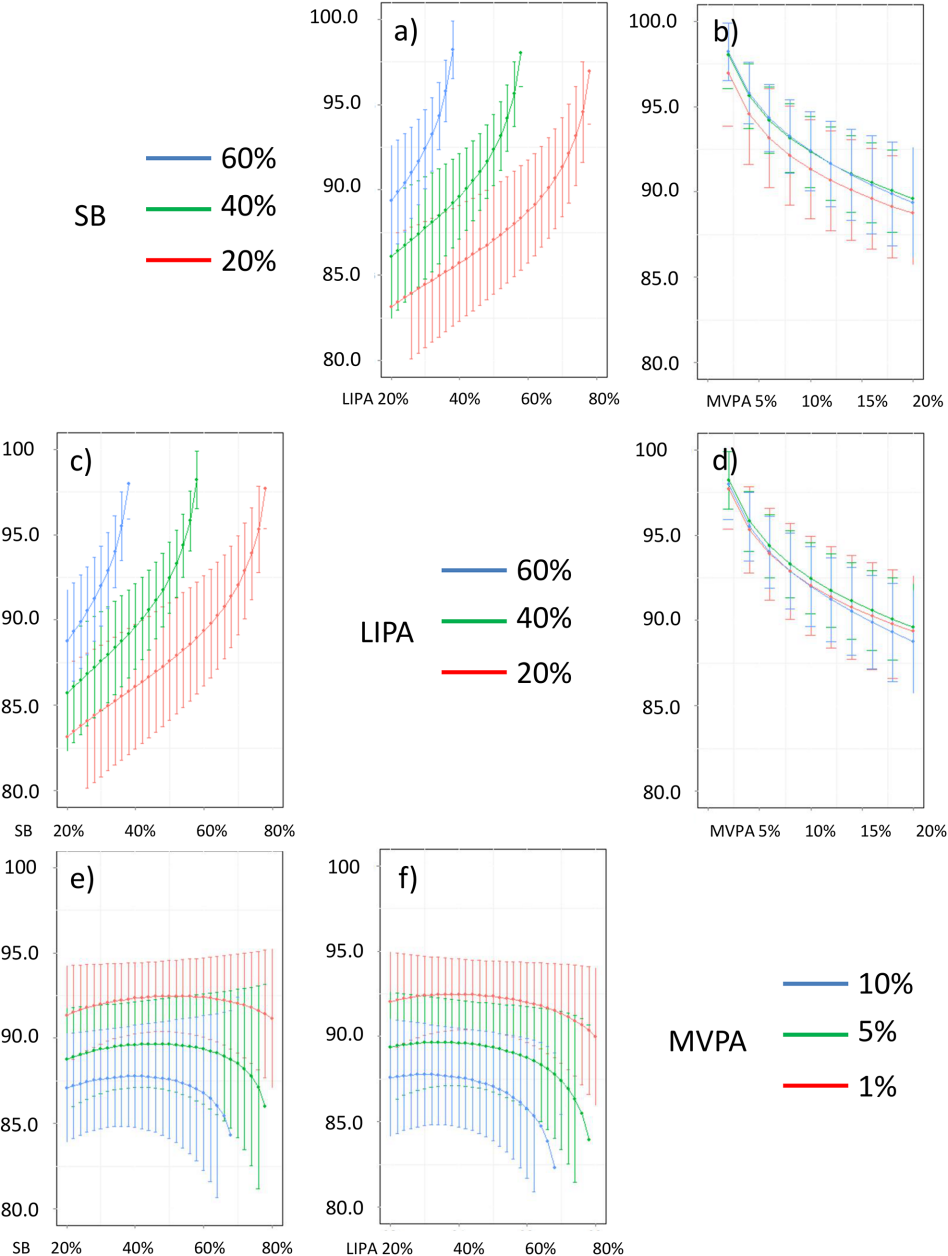
Estimated waist circumference (in cm) for different composition as a function the proportion of a) LIPA and b) MVPA when SB is held constant, c) SB and d) MVPA when LIPA is held constant, e) SB and f) LIPA when MVPA is held constant. The plots correspond to slices of Fig 11B.

**Fig 18:**
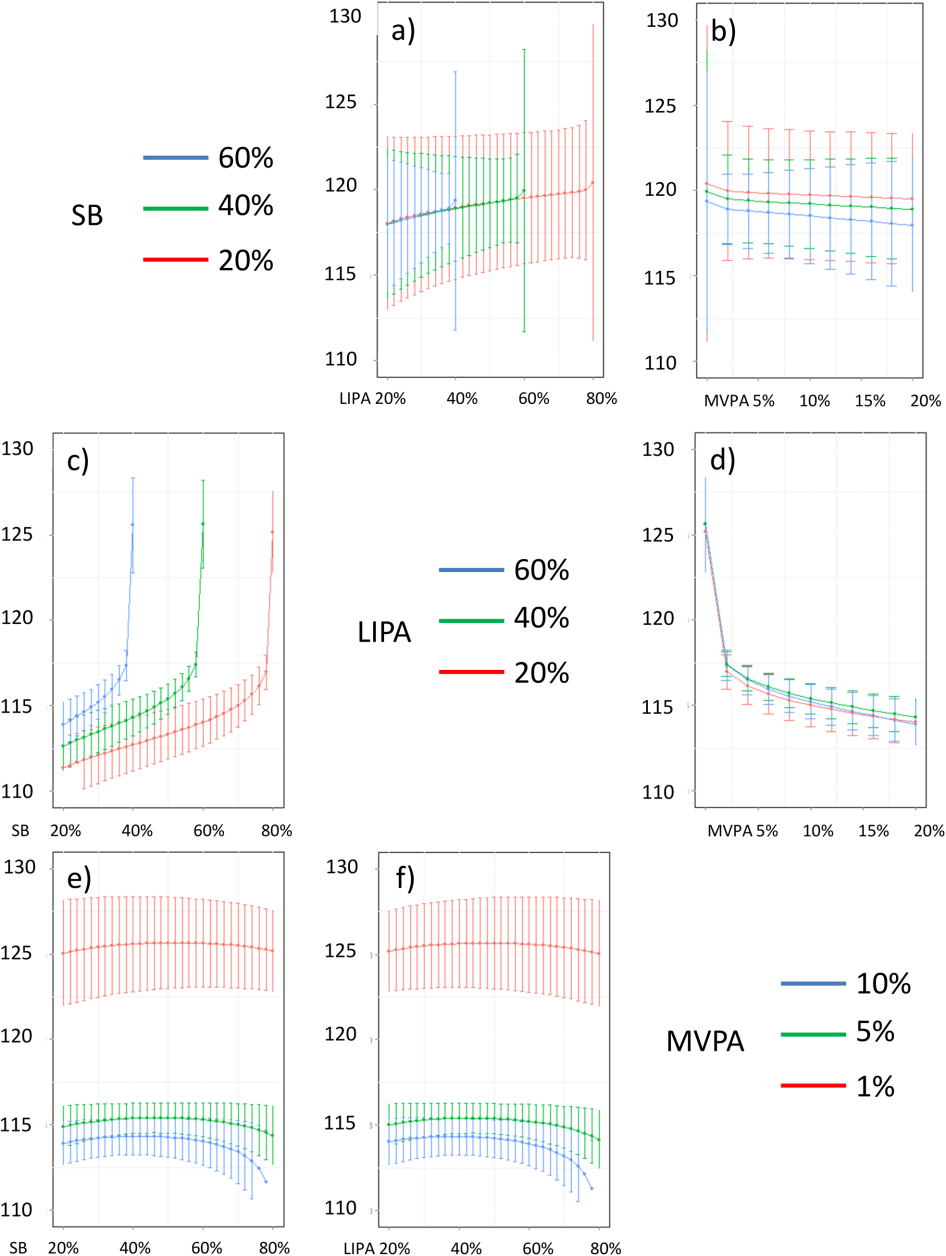
Estimated systolic blood pressure (in mmHg) for different composition as a function the proportion of a) LIPA and b) MVPA when SB is held constant, c) SB and d) MVPA when LIPA is held constant, e) SB and f) LIPA when MVPA is held constant. The plots correspond to slices of Fig 12A.

**Fig 19:**
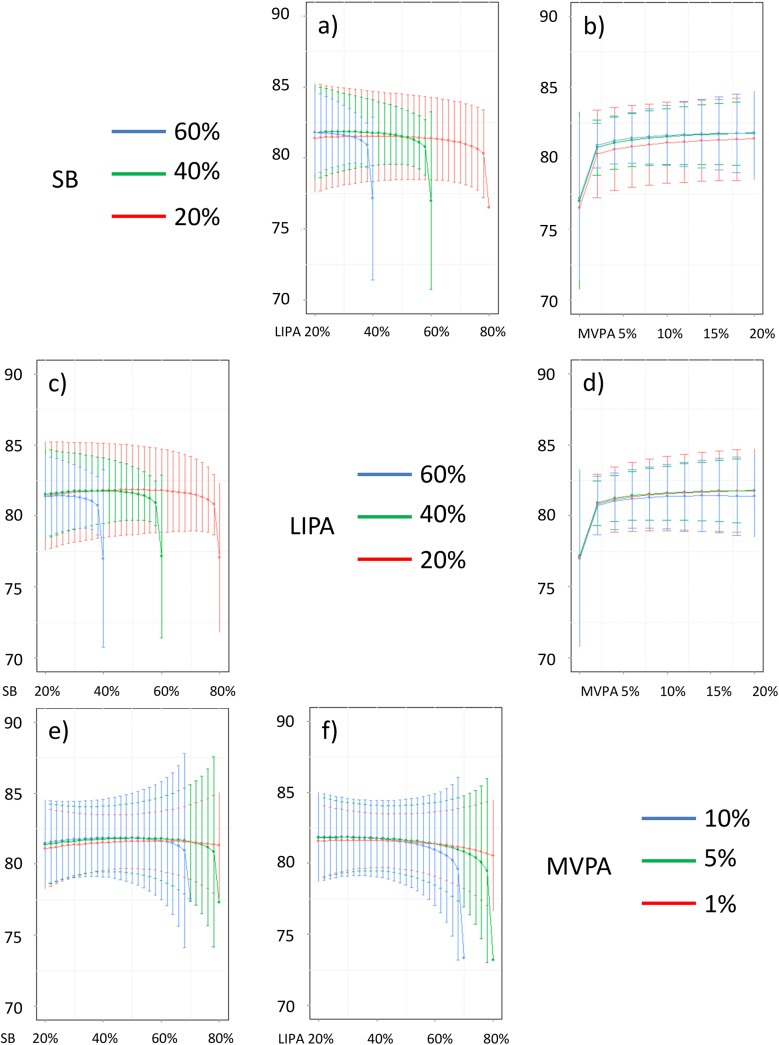
Estimated diastolic blood pressure (in mmHg) for different composition as a function the proportion of a) LIPA and b) MVPA when SB is held constant, c) SB and d) MVPA when LIPA is held constant, e) SB and f) LIPA when MVPA is held constant. The plots correspond to slices of Fig 12B.

**Fig 20:**
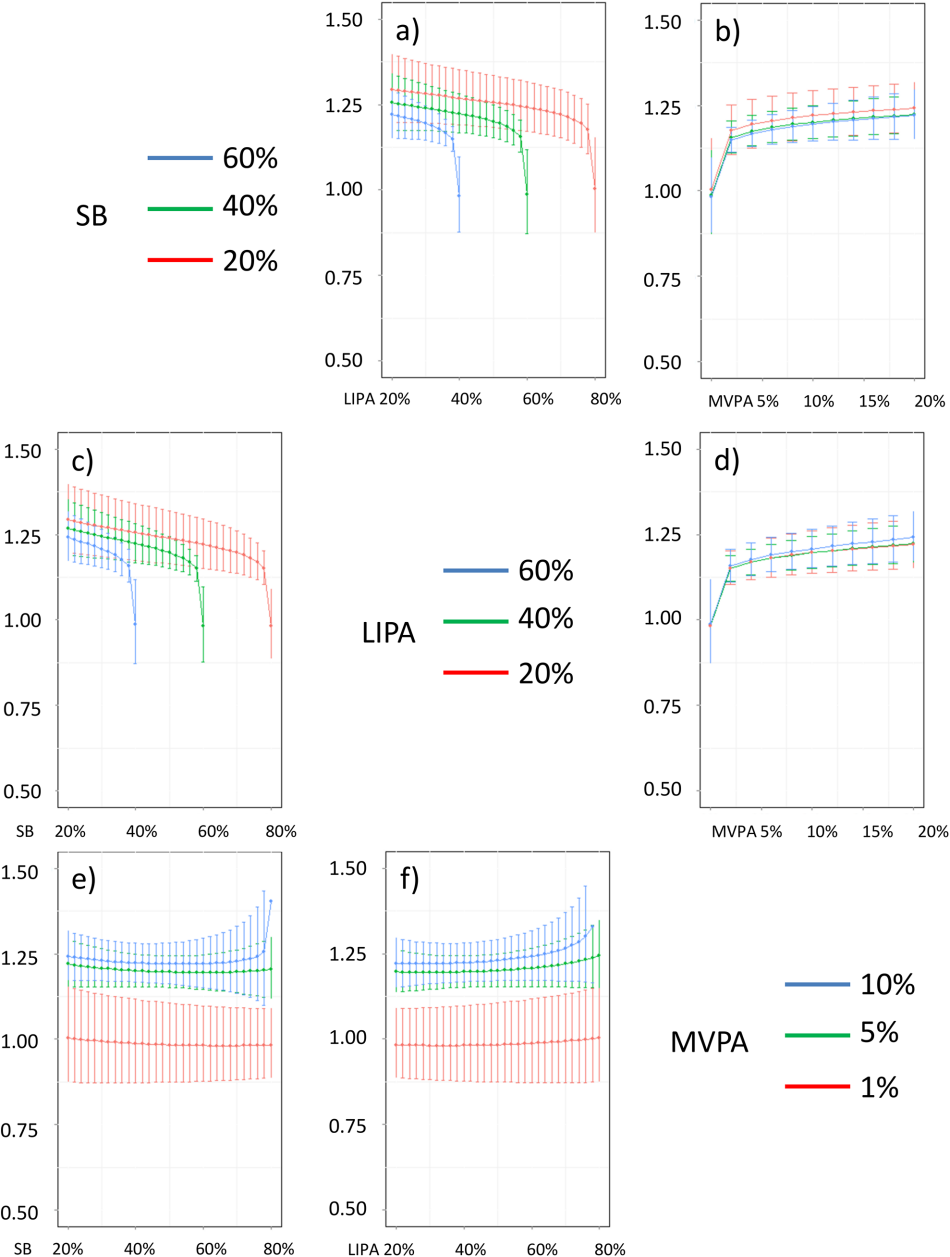
Estimated HDL (in mmol/L) for different composition as a function the proportion of a) LIPA and b) MVPA when SB is held constant, c) SB and d) MVPA when LIPA is held constant, e) SB and f) LIPA when MVPA is held constant. The plots correspond to slices of Fig 13A.

**Fig 21:**
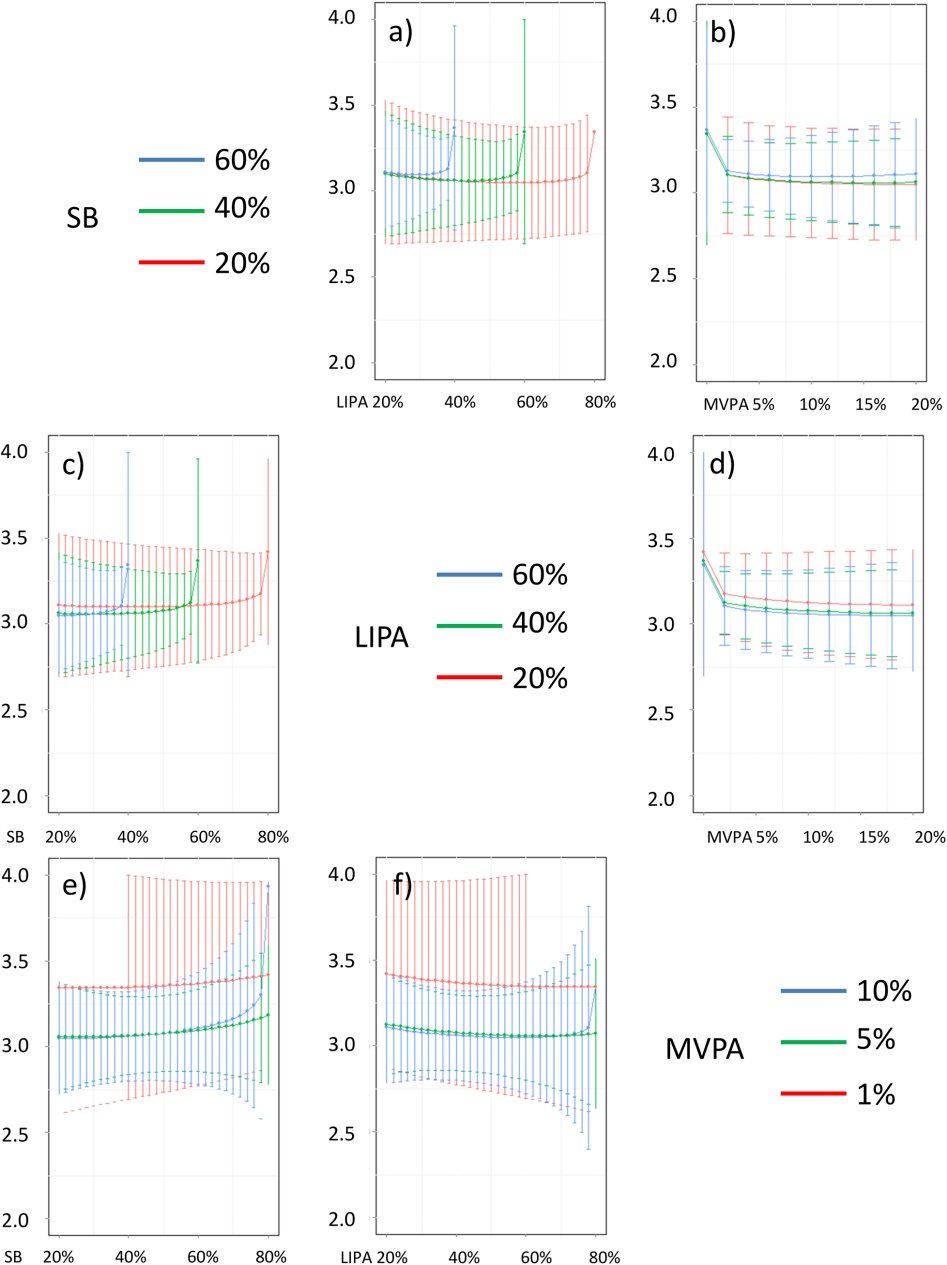
Estimated LDL (in mmol/L) for different composition as a function the proportion of a) LIPA and b) MVPA when SB is held constant, c) SB and d) MVPA when LIPA is held constant, e) SB and f) LIPA when MVPA is held constant. The plots correspond to slices of Fig 13B.

**Fig 22:**
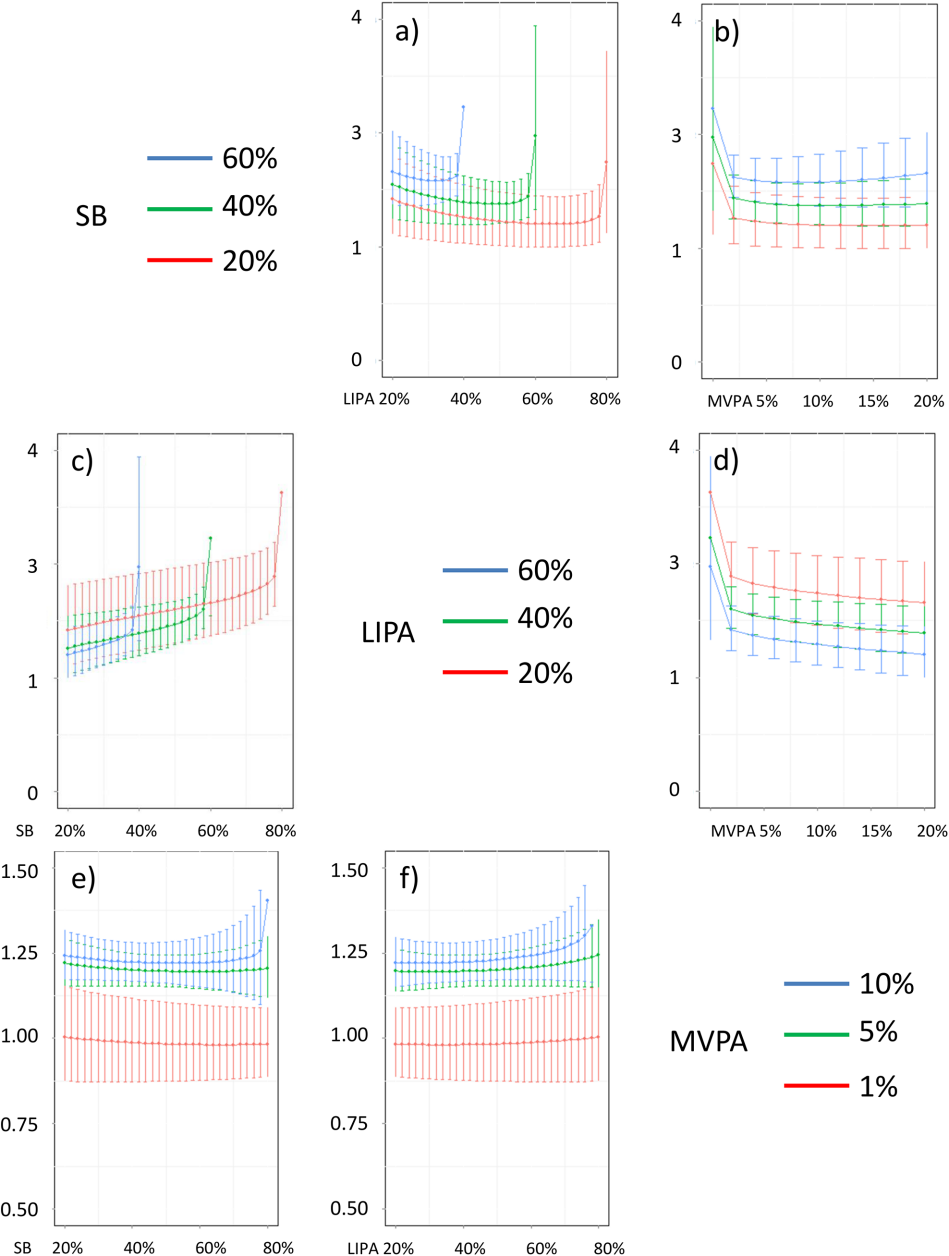
Estimated triglycerides (in mmol/L) for different composition as a function the proportion of a) LIPA and b) MVPA when SB is held constant, c) SB and d) MVPA when LIPA is held constant, e) SB and f) LIPA when MVPA is held constant. The plots correspond to slices of Fig 14A.

**Fig 23:**
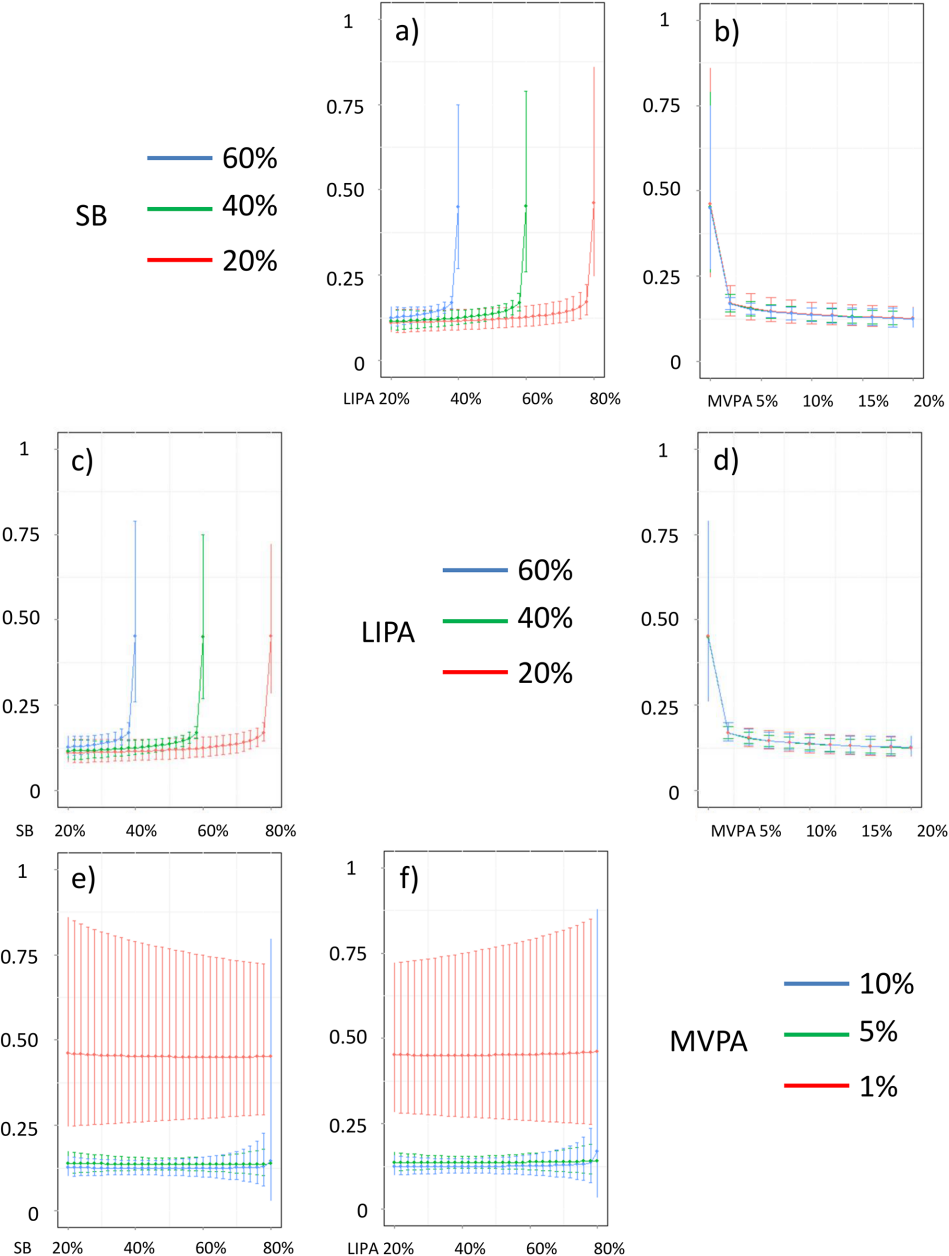
Estimated CRP (in mg/dL) for different composition as a function the proportion of a) LIPA and b) MVPA when SB is held constant, c) SB and d) MVPA when LIPA is held constant, e) SB and f) LIPA when MVPA is held constant. The plots correspond to slices of Fig 14B.

**Fig 24:**
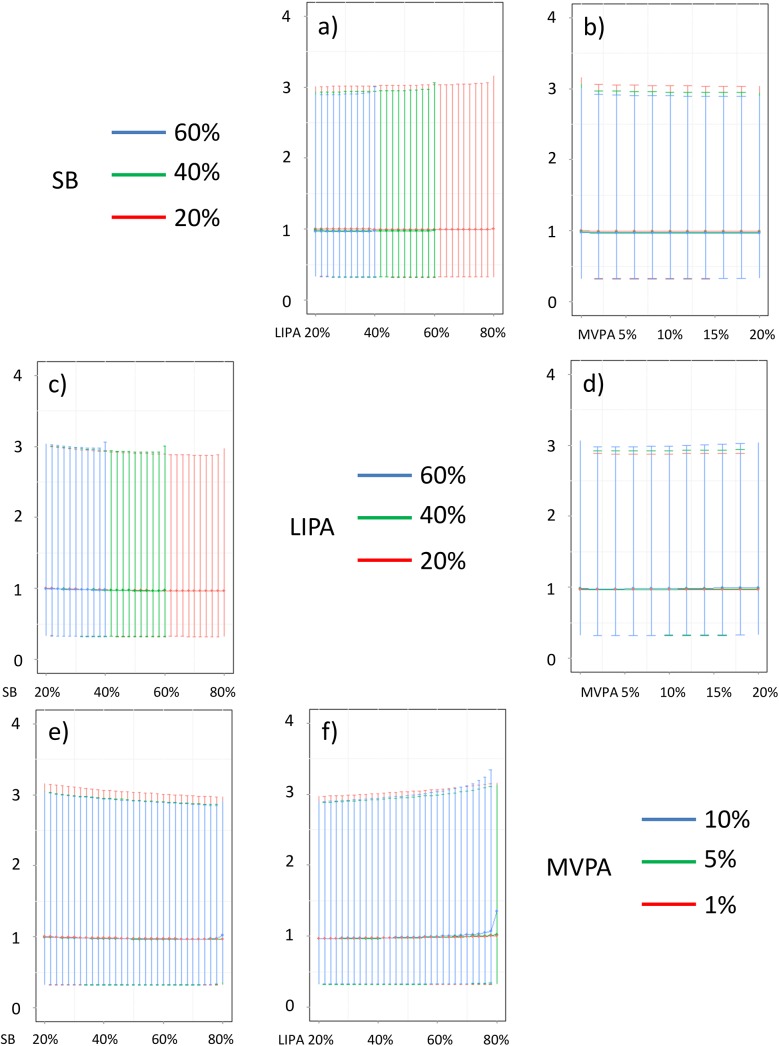
Estimated plasma glucose (in mmol/L) for different composition as a function the proportion of a) LIPA and b) MVPA when SB is held constant, c) SB and d) MVPA when LIPA is held constant, e) SB and f) LIPA when MVPA is held constant. The plots correspond to slices of Fig 15A.

**Fig 25:**
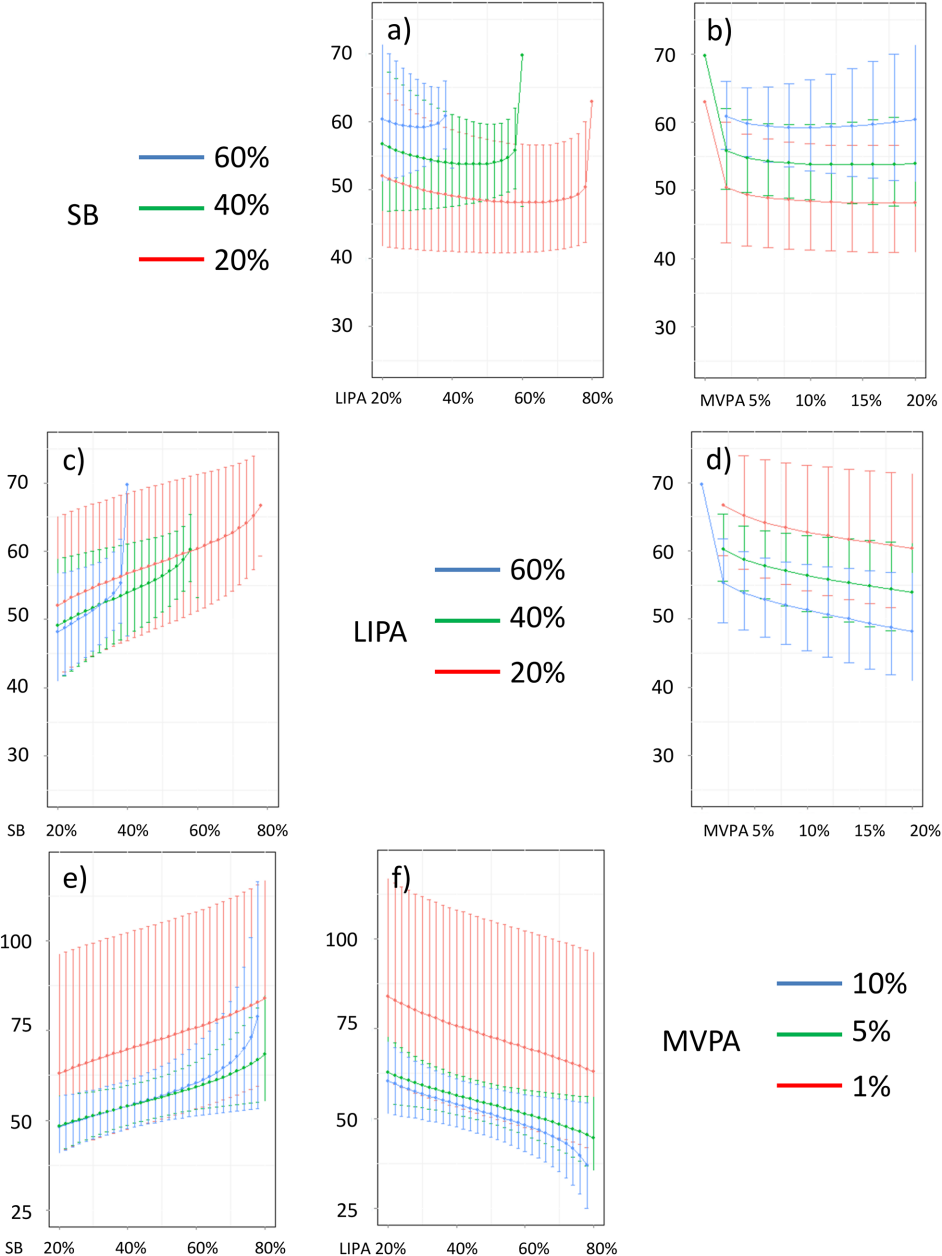
Estimated plasma insulin (in pmol/L) for different composition as a function the proportion of a) LIPA and b) MVPA when SB is held constant, c) SB and d) MVPA when LIPA is held constant, e) SB and f) LIPA when MVPA is held constant. The plots correspond to slices of Fig 15B.

**Fig 26:**
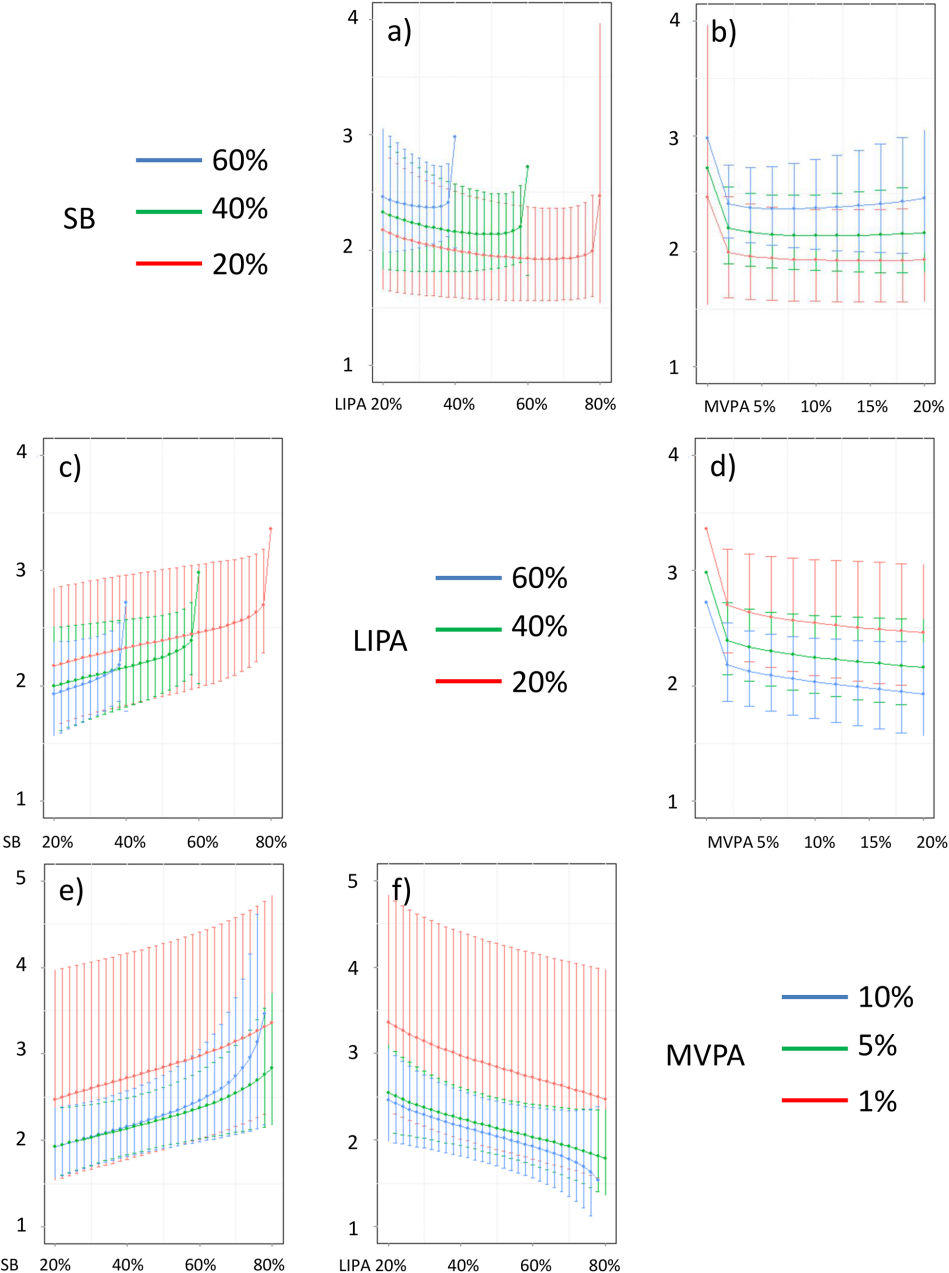
Estimated HOMA for different composition as a function the proportion of a) LIPA and b) MVPA when SB is held constant, c) SB and d) MVPA when LIPA is held constant, e) SB and f) LIPA when MVPA is held constant. The plots correspond to slices of Fig 15C.

In addition, Figs 16–26 represent slices of those ternary plots. In these graphs, the change in the outcome is plotted as a function of the proportion of time spent in two behaviors for a given proportion of time in the third one. For example, in Fig 16A each curve represents the difference in BMI as a function of the proportion of time spent in LIPA for a given proportion of SB. The increase in the proportion of LIPA time in this graph correspond to equivalent decreases of time in MVPA. The reciprocal plot showing differences in outcome as a function of the proportion of time spent in MVPA for a given proportion of SB is displayed in Fig 6B. Standard 95% confidence intervals of the prediction are shown as error bars in these graphs.

Estimations were computed for white men, aged 43, non-smoker, with BMI = 28.8 kg/m^2^, with an income to poverty ratio of 2.87 and college level education, consuming 24.9 mg of saturated fat daily, 11 mg of alcohol, 100 mg of caffeine, taking no blood pressure medication or diabetic drugs and self-reporting good health. The absolute values of these estimates varied depending on the value of the confounding variables. The trends observed however remained similar. Therefore these graphs should be interpreted as indication of the trend and relative magnitude of the difference in outcomes rather than in terms of their absolute values.

Taken together, these plots show that MVPA positively affects all outcomes, except glucose (Figs 15 and 24) when replacing either LIPA or SB time. The magnitude and trend appeared to vary depending on the proportion of time in LIPA and SB, especially for triglycerides (Figs 14 and 24), insulin (Figs 15 and 25) and HOMA (Figs 15 and 26), and to a lesser degree for waist circumference (Figs 11 and 17). The effect was relatively stronger at lower proportions of MVPA and weakened as the proportion of time spent on MVPA was higher, which is consistent with current evidence on dose-response [3]. Again this seemed to vary depending on the proportion of time in LIPA and SB and the cardiometabolic outcome considered.

The effects of light activity were less pronounced but appeared negative when replacing MVPA on BMI (Figs 11 and 16), waist circumference (Figs 11 and 17) and systolic blood pressure (Figs 12 and 18), and these were different depending on the proportion of time spent on SB for BMI and waist circumference, but not for systolic blood pressure. Weak negative effects were observable for HDL (Figs 13 and 20) and CRP (Figs 14 and 23), but these became stronger when LIPA replaced MVPA. These also depended on the proportion of SB. LIPA had positive effects most notably on insulin (Figs 15 and 25) and HOMA (Figs 15 and 26) and, to a lesser extent, on triglycerides (Figs 14 and 24). However these effects depended on the proportion of MVPA and were strongest when LIPA replaced SB (Figs 25F and 26F).

The proportion of time spent in SB had notable negative effects on BMI (Figs 11 and 16), waist circumference (Figs 11 and 17), systolic blood pressure (Figs 12 and 18), HDL (Figs 13 and 20), CRP (Figs 14 and 23), triglycerides (Figs 14 and 24), insulin (Figs 15 and 25) and HOMA (Figs 15 and 26) when it replaced MVPA time. However, the magnitude of these effects appeared to depend on the proportion of LIPA. No noticeable effects of SB time were observed when SB replaced LIPA time, except for insulin (Figs 15 and 25) and HOMA (Figs 15 and 26) and, to a lesser extend, triglycerides (Figs 14 and 24).

These results are for 8 hours of time spent sleeping. The magnitude of the effect varied when other proportions of sleep time were considered but the trends and observed co-dependences between waking day behaviors remained unchanged.

## Discussion

This study is the first to our knowledge to use a compositional analysis to provide a statistically sound and comprehensive investigation of the associations between the relative distribution of time spent in different physical behaviors that make up the day and cardio-metabolic health markers. In our view, this study addresses two important gaps in the literature. First, it contributes to mitigate the dearth of information about the combined effect of sleep, SB, LIPA and MVPA on health [14,27]. Second, it provides a solution to study the relationship between health and time budgets without encountering issues of spurious correlations and collinearity [8].

Recently, research on SB and LIPA in the physical activity research community has tended to revolve around two main issues: the strength of the association between health markers and time spent sedentary or in light activity and whether these associations are independent of the time spent active. Some authors claim that sedentary time has specific effects on health that are not mitigated by activity [5,28] whilst others consider it as inactivity together with LIPA which simply has an effect because it displaces active time [10,14,27]. While there is plenty of evidence for a deleterious association between SB and health [29–34] and some about the potentially beneficial effect of LIPA [7,35,36] it is not entirely unequivocal [6,37–40]. The debates revolves around whether or not epidemiological models are correctly adjusted for what happens during the rest of the day [8,10] and especially the confounding effects of MVPA and sleep time.

The fact that time is finite during the day strongly suggests that the debate on whether or not the effect of a single behavior is independent of another one might be conceptually wrong. Time spent in one behavior is naturally co-dependent of the time spent in others and the effect of time spent in one behavior should naturally depend on the composition of the rest of the day.

Our analysis inherently deals with this issue of relative time adjustment and shows that for most obesity and cardio-metabolic health markers (BMI, waist circumference, blood pressure, triglycerides, C—reactive protein, plasma insulin and HOMA) the composition of time spent in sleep, SB, LIPA and MVPA appears to matter as a whole. Indeed the composition is significantly associated with all markers except HDL and LDL. Within the composition, the proportions of time spent in MVPA and SB still show statistically significant associations with some markers (Table 3). For each behavior, the regression coefficients enable us to estimate their effect when the model is adjusted for the entire relative distribution of time. However, it is important to note that they should not be interpreted as an independent effect, as they correspond to the effect of the relative contrast of time spent in one behavior compared to the other three ones.

Table 4 shows that the strongest effects are observed when SB displaces MVPA. In addition, when the compositional model is compared to the single behavior models we found that the strength of association is greatly attenuated. This is in particular for MVPA, which suggests that the effect of MVPA is not entirely independent of SB, LIPA and sleep. The graphs in Figs 11–26 strongly suggest that the relationships between markers of cardio-metabolic health and each behavior are changed and moderated by time spent in the other behaviors.

Our analysis shows that each behavior might affect health both directly and also indirectly because it displaces the others. Teasing this out might not be as fruitful as trying to understand how different compositions affect markers and health outcomes.

**Fig 27:**
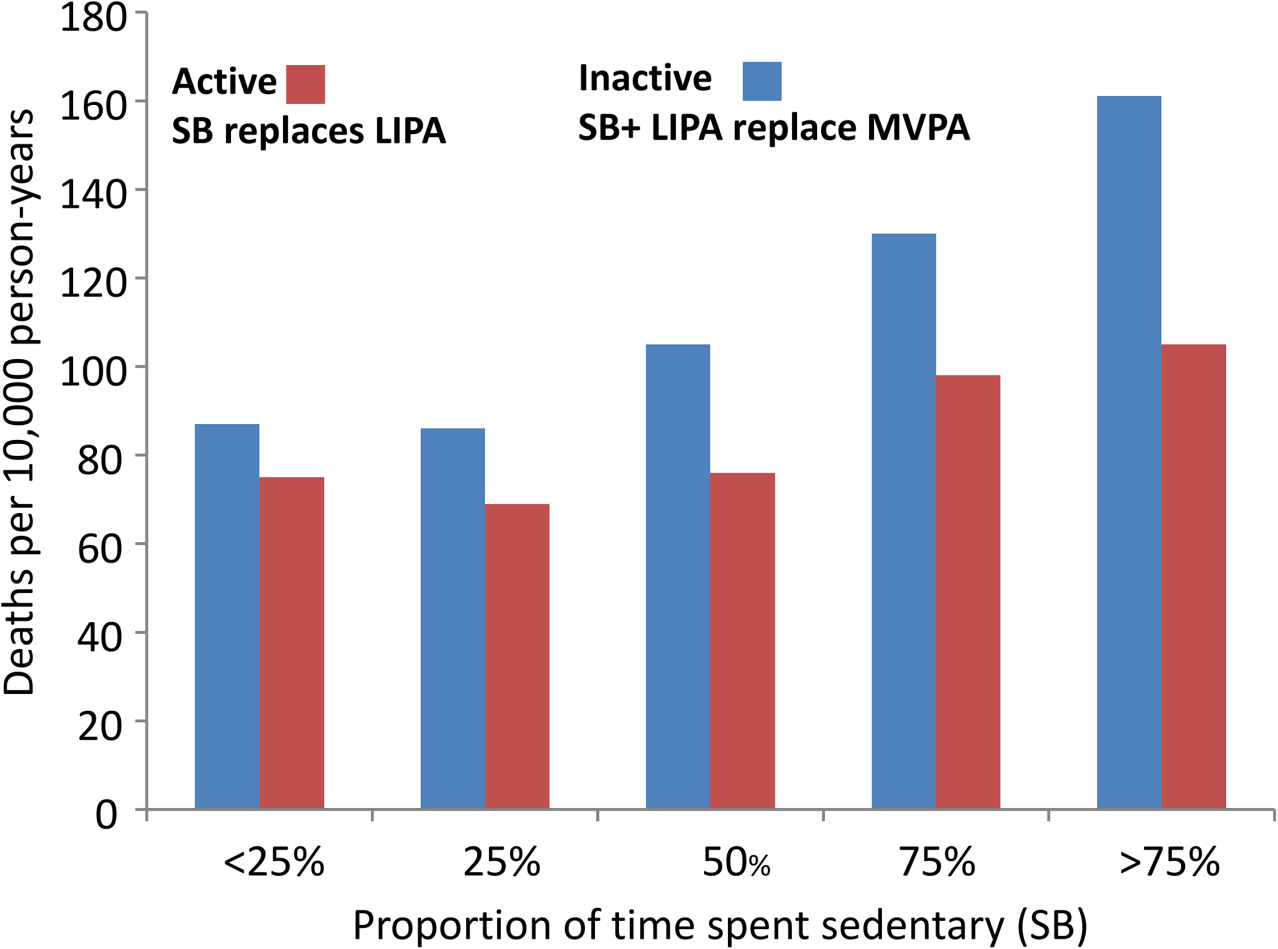
Mortality rate as function of proportion of time spent sedentary for active people and inactive people. Adapted from Katzmarzyk et al., 2009. MSSE. 41(5): 998–1005. The red bars show the effect of sedentary behaviors (SB) replacing light activity (LIPA) at equal proportion of time spent in moderate to vigorous activity (MVPA). The blue bars show the effect when SB + LIPA displace MVPA.

In our results, the relative times spent in SB and LIPA were both detrimentally associated with obesity markers, HDL, CRP and blood pressure, while MVPA showed a health enhancing relationship. This could suggest that inactivity (SB+LIPA) is detrimental and MVPA beneficial and that is all that matters. However the strength of the negative relationship was higher with SB than with LIPA, and in Table 4 the effect of replacing MVPA with SB was stronger than with LIPA. This suggests that LIPA might be a lesser “evil”. Humans cannot perform MVPA all day, therefore, although LIPA might be detrimental, spending the rest of the day in LIPA might be better than sitting (SB). Hence, for people who cannot engage in MVPA and exercise, or simply do not want to, promoting LIPA might be beneficial. Our result could explain why SB has appeared associated detrimentally with health seemingly independently of MVPA, as reported in mortality rate in [41]. The combined effect of LIPA and SB, when displacing MVPA is associated with higher health risk/mortality. But, at equal proportion of MVPA, if SB replaces LIPA the detrimental effect of inactivity could be higher, then explaining the added mortality for active people who sit for a long time (Fig 27).

For diabetes risk markers (insulin and HOMA) time spent in SB and LIPA had the opposite relationship, suggesting that replacing sitting time with light activity might be beneficial (Table 3, Figs 15B, 15C, 25F and 26F). This is in agreement with recent intervention studies [42]. In addition, replacing SB by LIPA also had as strong an effect as replacing it with MVPA (Table 4), as it has been observed experimentally [43–45]. A similar pattern was observed for triglycerides. In this respect, our results provide epidemiological evidence that the acute effects of replacing SB with LIPA observed in experimental study might translate to longer-term benefits for glycaemic control. All combined, this evidence suggests that substituting sitting time for light everyday activities might contribute to preventing and managing diabetes.

Our results do not disagree with the overwhelming evidence that MVPA is beneficial to health, but the analysis shows that this relationship depends on the composition of the rest of the day. A striking result was that the effect of substituting MVPA with other behaviors was not the same if the proportion of time in MVPA is increased or decreased. For example, replacing 10 minutes of MVPA with 10 minutes of SB corresponded to a 1.2% higher BMI, while the opposite, replacing 10 minutes of SB with 10 minutes of MVPA, had a 1000 fold lower effect. This is in stark contrast with results using isotemporal substitution with this same data set [14] and others [27] which reported much higher and symmetric effects. Our results are supported by two facts. Firstly, removing 10 minutes from MVPA is actually a large amount of MVPA equal to one third of the recommended guidelines [2], while decreasing SB time by 10 minutes only accounts for a 2 to 5% change in total SB time. Therefore, a symmetric effect does not seem realistic. Secondly, it is also well known that deconditioning or weight gain occurs rapidly when activity levels are dropped, and that it takes a larger amount of exercise overload to return to the same weight or fitness [46–48].

Sleep in our analysis was directly beneficially associated with obesity and blood pressure markers but could have negative effects if it displaced MVPA. This is also consistent with current literature [49]. At high and low proportions, sleep also appears to act as a moderator of the effect of the waking day behaviors, enhancing negative associations for sleep time over 7 hours. Sleep was also found as deleteriously associated with triglycerides, CRP and plasma glucose level. This was not reported previously by analysis of this wave of NHANES [14] but it is consistent with evidence that long sleep duration are generally associated with worse health outcomes [50–53]. The literature is not clear about the reason for this association. It might be a direct consequence of physiological mechanism occurring during long sleep, but also could be due to the fact that people who report sleeping for a long time actually stay in bed longer and experience more disrupted and poorer quality sleep [54].

Our results suggest that public health messages should target all physical behaviors synergistically to maximise health, and that interventions aimed simply at maintaining MVPA level might have a more profound effect than previously thought. Indeed, we found that a small decrease in MVPA time has a large effect while an increase in MVPA has more moderate effects. Most interventions or public health campaigns are aimed at increasing physical activity, but very few specifically aim at supporting people to remain active through the life course and throughout seasonal changes. Maintaining activity levels of the population could translate to net health and economical benefits.

While a compositional approach could be seen as a mere methodological issue, it actually constitutes a radical change in the way we conceptualize activity through the day. A compositional paradigm opens the door to finding the optimum distribution of time spent in different physical behaviors throughout the day and to integrated guidelines for activity, sedentary behaviors and sleep that could be mapped as in Figs 11–15.

Here we have applied a compositional paradigm to the study of associations between health and daily activity via linear regression, but more far-reaching compositional methods could be integrated easily into most types of analyses and study designs. For example sub-classes of sedentary behaviors such as screen-time [55] could be considered, or the partition of the day could be based on postural allocation (lie, sit, stand, walk, run) rather than energy to evaluate the effect of intervention or longitudinal studies. Twenty four hour monitoring protocols, during which monitors are worn continuously, are becoming more common. This will remove issues with non-wear time. In addition, it is likely that detection of sleep time will improve in the future as well as the detection of other subclasses of behavior. This improved accuracy, coupled with compositional analysis, could potentially lead to greater insight. However, applying compositional analysis is not restricted to 24-hour data, but actually applies for any time budget comprising any segment of the day and whether this is assessed objectively or subjectively. Indeed one of the main principles of compositional data is that results do not depend on the total nor on whether or not we are working with the full composition [11] (for further details see S2 File).

The strength of this study resides in the compositional analysis applied to a well-known and characterised data set, in which activity and sedentary behaviors are objectively measured. Objective measurements are less prone to error [56], especially for quantifying time spent in MVPA. This is however less true for SB and LIPA time. The accelerometer data were classified every minute of the day as MVPA, SB or LIPA depending on a threshold. This method is less accurate than postural allocation at distinguishing between SB and LIPA [57], therefore it is possible that these two components of the composition might not be entirely accurate and that different thresholds might lead to different results. Another limitation is that, unfortunately, objective data for sleep time were not available. In addition, sleep was only evaluated over week or working days, while the accelerometry was averaged over the whole week. It is likely that this introduced some random error. It is known that activity patterns can be different at weekends or non-working days. Similarly sleep duration varies at weekends or non-working days depending on chronotypes. The NHANES sample includes people with different working shifts and both employed and unemployed individuals. Unfortunately, the NHANES data do not allow reliable isolation of the non-working days or information about chronotype.

The data loss from the full NHANES sample is relatively high (around 50% see S1 File) due to compliance with objective monitoring and this selection bias affects the representativity of the sample analyzed.

Finally, as with all cross-sectional analysis, causal inference is limited and the estimated effects reflects more a population shift in distribution of time rather than actual effects for individuals.

## Conclusion

In summary, time spent on a physical behavior is co-dependent on the other ones and, therefore, it should be analysed and conceptualised within a compositional paradigm to obtain meaningful and accurate inferences. Our analysis adds to the current evidence that decreasing inactivity, in particular sedentary time, and maintaining or increasing time spent in moderate to vigorous physical activity, contributes toward a more favorable cardiometabolic risk profile. However, the health risks are higher when more of this inactive time is spent in SB rather than in LIPA, even with the same amount of time spent in MVPA. Therefore, it is important to prevent a transfer of time from LIPA to SB. It is also important to prevent a transfer of time from MVPA to LIPA or SB. Although replacing SB by MVPA has a stronger positive health effect, our results show that replacing SB by LIPA could have beneficial health effects as well.

## Supporting Information

S1 File Study characteristics. (DOCX)

S2 File Concise guide to compositional analysis for physical activity, sedentary behaviors and sleep research. (PDF)
