# Supplementary material for: Combined Effects of Time Spent in Physical Activity, Sedentary Behaviors and Sleep on Obesity and Cardio-Metabolic Health Markers: A Novel Compositional Data Analysis Approach
Source: PLoS One. 2015 Oct 13;10(10):e0139984. doi: 10.1371/journal.pone.0139984 (PMC4604082; doi:10.1371/journal.pone.0139984)
Supplement: S1 File — (DOCX) [file pone.0139984.s001.docx]

**Supplementary material S1**

Table A: Sample characteristics

| Characteristic | Eligible sample | Analysed sample | Test for difference |
| --- | --- | --- | --- |
| N | 3688 | 1937 |  |
| Age (median , [IRQ] years) | 40 [29-51] | 43 [32-54] |  |
| Age distribution (%) |  |  | p=0.199 |
| 21-39 | 52.3 | 44.1 |  |
| 40-59 | 39.7 | 45.6 |  |
| 60-64 | 8.0 | 10.3 |  |
| Gender distribution (%) |  |  | p=1.000 |
| Male | 46.9 | 49.0 |  |
| Female | 53.1 | 51.0 |  |
| Race/Ethnicity distribution (%) |  |  | P=0.220 |
| Mexican american | 23.0 | 23.3 |  |
| Other hispanic | 3.5 | 2.8 |  |
| Non-hispanic white | 45.0 | 47.4 |  |
| Non-hispanic black | 23.8 | 21.9 |  |
| Other and mixed race | 4.7 | 4.6 |  |
| Education level distribution (%) |  |  | p=0.199 |
| Less than 12 years | 47.5 | 44.8 |  |
| 12 years | 30.8 | 31.5 |  |
| Over high school | 21.7 | 23.7 |  |
| Marital status distribution (%) |  |  | P=0.220 |
| Married | 55.7 | 61.1 |  |
| Widowed | 2.2 | 2.3 |  |
| Divorced | 9.9 | 10.3 |  |
| Separated | 3.2 | 3.0 |  |
| Never married | 18.6 | 15.0 |  |
| Living with partner | 10.4 | 8.3 |  |
| Income to poverty ratio distribution (%) |  |  | P=0.213 |
| 0-1.20 | 23.0 | 19.6 |  |
| 1.21-2.28 | 22.0 | 21.6 |  |
| 2.29-4.11 | 25.5 | 27.2 |  |
| ≥4.12 | 29.5 | 31.6 |  |
| BMI (median [IQR]) kg/m^2^ | 27.8 [24.3-32.3] | 27.8 [24.5-32.0] | p=0.242 |
| Waist circumference (median [IQR]) cm | 96.5 [86.2-107.3] | 96.3 [86.6-107.3] | P=0.213 |
| Systolic Blood Pressure (median [IQR]) mmHg | 117.3 [109.3-128.0] | 117.3 [109.3-128.7] | P=0.213 |
| Diastolic Blood Pressure (median [IQR]) mmHg | 70.0 [62.7-76.7] | 70.7 [64.0-77.3] | P=0.261 |
| HDL (median [IQR]) mmol/L | 1.34 [1.09-1.66] | 1.34 [1.09-1.66] | P=0.213 |
| LDL (median [IQR]) mmol/L | 2.95 [2.33-3.60] | 3.05 [2.38-3.62] | P=0.213 |
| Triglycerides (median [IQR]) mmol/L | 1.29 [0.88-1.99] | 1.29 [0.88-1.99] | P=0.238 |
| C-Reactive protein (median [IQR]) mg/dL | 0.22 [0.08-0.54] | 0.21 [0.08-0.49] | P=0.238 |
| Plasma glucose (median [IQR]) mmol/L | 5.32 [4.94-5.77] | 5.38 [5.05-5.77] | P=0.213 |
| Plasma insulin (median [IQR]) pmol/L | 52.7 [32.8-90.2] | 51.7 [33.3-87.1] | p=0.915 |

Covariates: Models were adjusted for ethnicity/race, gender, marital status, family income to poverty ratio, educational level. Difference between analysed and eligible sample were tested using Chi-Square test.

Table B: Covariates used in the linear regression for each cardio-metabolic outcome

| Outcome | Confounders |
| --- | --- |
| BMI,WAIST | Age, gender, alcohol intake, dietary energy intake, dietary saturated fat intake, self-reported health and number of medical conditions |
| SBP,DBP | Age, gender, race/ethnicity, income to poverty ratio, alcohol, smoking status, self-reported health, educational level, use of blood pressure drugs |
| Log(HDL),LDL | Age, gender, race/ethnicity, dietary saturated fat intake, alcohol intake, smoking status, self-reported health, educational level |
| Log(TRI) | Gender, race/ethnicity, income to poverty ratio, marital status, smoking status, dietary caffeine intake, educational level, use of blood pressure drugs, BMI |
| Log(crp) | Age, gender, race/ethnicity, dietary saturated fat intake, income to poverty ratio, dietary energy intake, dietary caffeine intake, number of medical condition, self-reported health, educational level, BMI |
| Log(glu) | Age, gender, race/ethnicity, dietary saturated fat intake, dietary energy intake, dietary caffeine intake, use of diabetic drugs, educational level, BMI |
| Log(ins) | Age, gender, race/ethnicity, marital status, dietary energy intake, dietary caffeine intake, self-reported health, use of blood pressure and diabetic drugs, BMI |
| Log(homa) | Age, gender, race/ethnicity, marital status, dietary energy intake, dietary caffeine intake, alcohol intake, smoking status, self-reported health, use of blood pressure and diabetic drugs, BMI |
| Log(homab) | Age, marital status, dietary energy intake, dietary caffeine intake, alcohol intake, self-reported health, use of blood pressure and diabetic drugs, BMI |


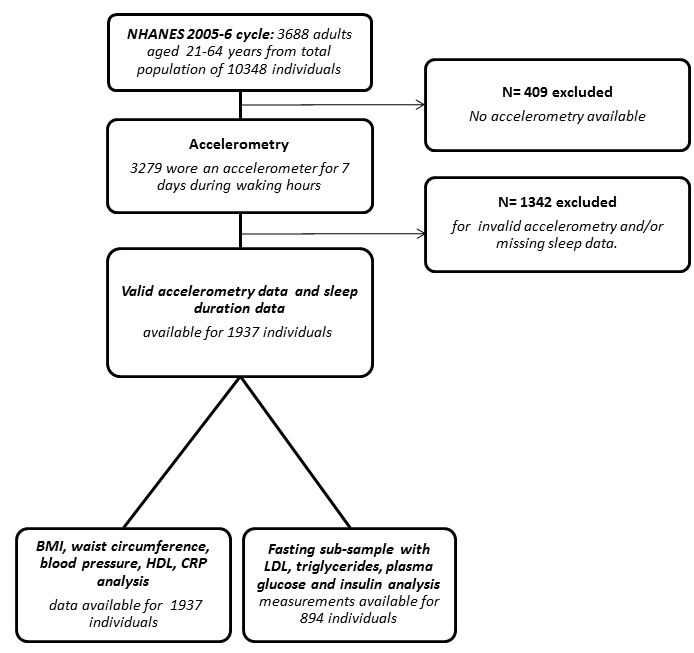


Figure A: Data flow chart
