# Supplementary material for: Combined Effects of Time Spent in Physical Activity, Sedentary Behaviors and Sleep on Obesity and Cardio-Metabolic Health Markers: A Novel Compositional Data Analysis Approach
Source: PLoS One. 2015 Oct 13;10(10):e0139984. doi: 10.1371/journal.pone.0139984 (PMC4604082; doi:10.1371/journal.pone.0139984)
Supplement: S2 File — (PDF) [file pone.0139984.s002.pdf]

SUPPLEMENTARY MATERIAL S2: CONCISE GUIDE TO COMPOSITIONAL DATA ANALYSIS FOR PHYSICAL ACTIVITY, SEDENTARY BEHAVIOR AND SLEEP RESEARCH

Authors: S Chastin, J. Palarea-Albaladejo
Date: 28/01/2015
Version:2.2

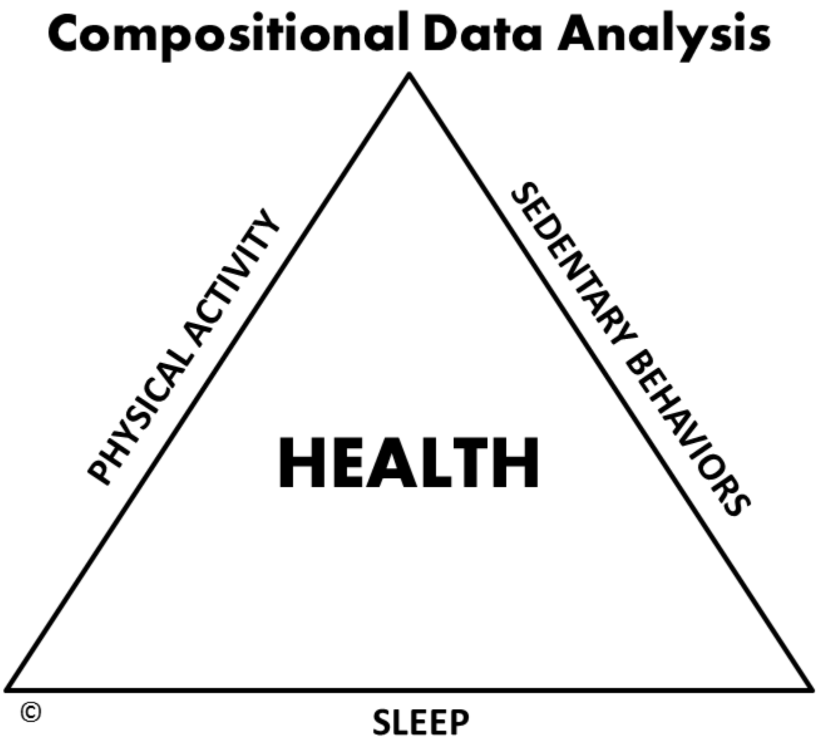

DISCLAIMER:

This document is a brief guide to the basis of Compositional Data Analysis (CoDa) intended to help researcher in physical activity, sedentary behavior and sleep epidemiology familiarize themselves with basic concepts and develop basic analysis. It is not a formal introduction to the topic. Researchers wishing to develop further understanding and who seek further information should use the references at the end of this document.

Introduction

This concise guide is provided as supplementary material to the research article Chastin SFM, Palarea-Albaladejo J, Dontje ML, Skelton DA. "Combined effects of time spent in physical activity, sedentary behavior and sleep on adiposity and cardiometabolic health markers: a novel compositional data analysis approach.". 2015 XXXXXXX.

Please cite this manual as

Chastin SFM, Palarea-Albaladejo J. Concise Guide to Compositional Data Analysis for Physical Activity, Sedentary Behavior and Sleep Research: Supplementary Material S2, in Chastin SFM, Palarea-Albaladejo J, Dontje ML, Skelton DA. "Combined effects of time spent in physical activity, sedentary behavior and sleep on adiposity and cardiometabolic health markers: a novel compositional data analysis approach.". 2015 XXXXXXX.

Background

Population data is necessary to explore the relationship between the physical activity behavior human undertake during the day (sleep, sedentary behavior, physical activity – light, moderate and vigorous) and health. Most epidemiological studies are observational in nature as this is the most practical methods of obtaining data at a population scale. Much of physical activity behavior epidemiology uses regression analysis to investigate the variation of observed health outcomes that can be attributed to exposure to time spent in different behaviors. To date, the time allocated to each of these behaviors and its relationship to health has been studied in isolation [1]. However, we know very little about the combined effect of allocating time to these different behaviors. This dearth of information is due to the limitation of the standard multiple regression technique to deal with multivariate data that represent portions of a finite whole (such as a finite time period).

The underlying assumptions made in linear regression (and other variants of it such as logistic regression, ANOVA, etc.) imply that time spent in a behavior can be independent of the time spent in any other one and that it is potentially infinite. However the total time available in, say, a day is finite, hence time allocated to one behavior can neither be spent in another one nor be infinite. Data quantifying time spent in physical activity behaviors by nature violate the basic assumptions of linear regression; the times spent in different behaviors are intrinsically co-dependent, finite and subject to collinearity. While this might be considered merely as a technical difficulty, it has fundamental consequences. Linear regression with such finite and collinear data (even when apparently unrelated) can provide misleading results, with some effects being over or under estimated and some genuine effects obscured [2]. Thinking about time spent in different physical activity behaviors in isolation or as independent variables is nonsensical, essentially flawed, limits progress in epidemiological research and cast a shadow of doubt over current evidence.

Ways to properly conceptualize and analyze data with such characteristics have been investigated and discussed in the statistical literature [3,4] and it is nowadays an active area of methodological

research. This has been formally called compositional data analysis, and physical activity behavior data (collected over 24 hours or during part of the day) are essentially data of this kind, they are positive amounts representing parts of a finite whole (24 hours or length of the recording time). The main assumption an analyst makes when adopting a compositional approach to data analysis in our context is that the relevant information is in the relative distribution of time between behaviors, and not in their absolute values. That is, the amount of time spent on a behavior is meaningful only in light of the time spent on other behaviors and not on its own.

COMPOSITIONAL DATA ANALYSIS

Compositional data analysis is a well-established branch of statistics, which stems from Karl Pearson's work on spurious correlation [2] by the end of the nineteenth century. It has been mostly developed from the seminar monograph by John Aitchison in the 1980's [3]. It deals with data that represent parts or portions of a finite total, a mixture or composition in short. This type of data is usually closed (normalized) or re-scaled in order to add up to 1, and then work in proportions, or to 100, and then work in percentages. Note that the total is in fact irrelevant. Paradoxically, although this operation is very common in practice and in some way reflects the implicit interpretation of the data as relative amounts by practitioners, this feature is not further considered in the subsequent data analysis when using standard methods. So, for example, for a composition with d components x_i expressed in percentages

$$\sum_{i=1}^{d} x_i = 100\%.$$

DEFINITION IN THE CONTEXT OF PHYSICAL ACTIVITY BEHAVIOR

Physical activity behavior data are essentially compositional. For example body-worn sensors now enable us to measure precisely the time spent sleeping, in sedentary behavior (SB), in light activity (LIPA) or in moderate and vigorous activity (MVPA) over 24 hours. Hence the sum of the time spent in each behavior will be 24 hours, apart from slight measurement or rounding-off errors, and in percentage it will sum up to 100% of the day. For example, a 4-part composition consisting of sleep, SB, LIPA and MVPA times over a day would satisfy

$$t_{sleep} + t_{SB} + t_{LIPA} + t_{MVPA} = 24\ hours$$

This would still be true if we break down into sub-behaviors. For example we could have data about screen-based SB and non screen-based SB as well as leisure and occupational MVPA.

$$t_{sleep} + t_{SB_{screen}} + t_{SB_{non-screen}} + t_{LIPA} + t_{MVPA_{leisure}} + t_{MVPA_{occupational}} = 24\ hours$$

Other possible partitions could be based on posture, such as lie, sit, stand, walk, run, etc. We can generalize this further to any partition of the day into a set of d behaviors b_i

$$\sum_{i=1}^{d} b_i = 24\ hours$$

This is true of any body-worn sensor data, but also of subjective measures that record time spent in specific behaviors either through a single tool (e.g. IPAQ) or by combining several tools (e.g. IPAQ + sleep time diary + SBQ).

Very often however, we do not have the luxury of having 24-hour records, but instead we have data for only part of the day. This is commonly the case with accelerometry data, in which wear-time is an issue. While there is an increase toward 24-hour protocols in data collection, we do not always control the total time over which we have valid records. It is also common to not record all the behaviors, but instead a subset. For example, data may be available only for TV time, exercise time, transportation time but not for time spent in sleep, LIPA and non-screen SB. In both cases the total amount may not be 24 hours and may not even be the same across all individuals. Finally, these may all be measured in different units of time such as minutes, % of the day or % wearing time. It is then important to note that in all cases the data still carry relative information. The total time is irrelevant and the relative structure, given by the ratios between behaviors, remains the same regardless of the scale and of whether the observed subset of behaviors is closed or not to a same total time. As long as we can transform the units into each other, for example from minutes to hours or percentage, the compositional approach guaranties equivalent results in all cases.

GRAPHICAL REPRESENTATION OF COMPOSITIONAL DATA

The heart of the problem with physical activity behavior compositional data is that they are commonly treated as continuous numerical data defined on the standard real space. That is, multivariate data in which each component can freely vary in the interval $[-\infty, +\infty]$. However, the non-negativity and constant-sum constraints of compositional data imply that they are actually not well defined in this way.

For example, let's consider a 3-part composition made of SB, LIPA and MVPA time. If we wanted to plot the percentage time spent in each of these behaviors we are most likely to plot them on a 3-dimensional Cartesian coordinate system as shown in Fig Aa. Under the standard geometry of the real space, we assume that we can go from point A to B. That is, we assume that we can change the percentage of MVPA in this example, without actually changing the percentages of time spent in the other two behaviors, or that we can even reach point C which is beyond 100% MVPA. However, this is clearly not possible.

The fact is that compositional data actually live in the equilateral triangle represented in Fig. Ab in grey color. Note that a different constant sum, say 1 or 24, would only produce an equivalent triangle. Geometrically speaking, that triangle defines a constrained space called a simplex. This space is closed and therefore any change in MVPA affects either SB or LIPA, or both at the same time. Fig. Ac is obtained by projecting the triangle in Fig. Ab onto a 2-dimensional plane, and it is commonly known as ternary diagram or ternary plot. This has become the standard graphical tool to

visualize 3-part compositional data sets, in a similar way as the standard scatterplot is used to represent pairs of real (unconstrained) variables. The vertices represent the three behaviors of the composition; points which lie close to a vertex have high percentages of the behavior that is represented by that vertex, whereas points lying in the center of the triangle have equal percentages of all three behaviors. Each side of the triangle can be used as an axis representing values for each behavior, ranging from 0 to 100 in the case of percentages. A 3-dimensional representation of a 4-part composition based on a pyramidal arrangement is also possible, however this is hardly used in practice as the visualization of patterns is not usually very good.

Moving from one point to another on a ternary diagram accounts for the transfer of time from one behavior to other ones. It is important to note that, due to this intrinsic trade-off, translation of points, curves and other geometrical objects do not look on a ternary diagram as they do on standard graphs for real-space data. For example, Fig. B illustrates some operations and geometrical object on the simplex. Fig. Ba shows the linear translation of two points to a region closer to the bottom left vertex. Firstly, the distance between the two points (segment joining them) does not look like an ordinary straight line; a curvy line connects them instead. Secondly, even though the distance is maintained constant by translation, it is visually deformed as we move towards the boundaries of the triangle. Fig. Bb represents concentric circles at different locations on the simplex and we can appreciate how proximity to the boundaries of the triangle deforms iso-distance curves. Finally, in Fig. Bc we can see a cloud of points with a fitted linear regression curve passing through the center of the data cloud (big filled point), along with some iso-probability ellipses (probability regions) from the center of the data.

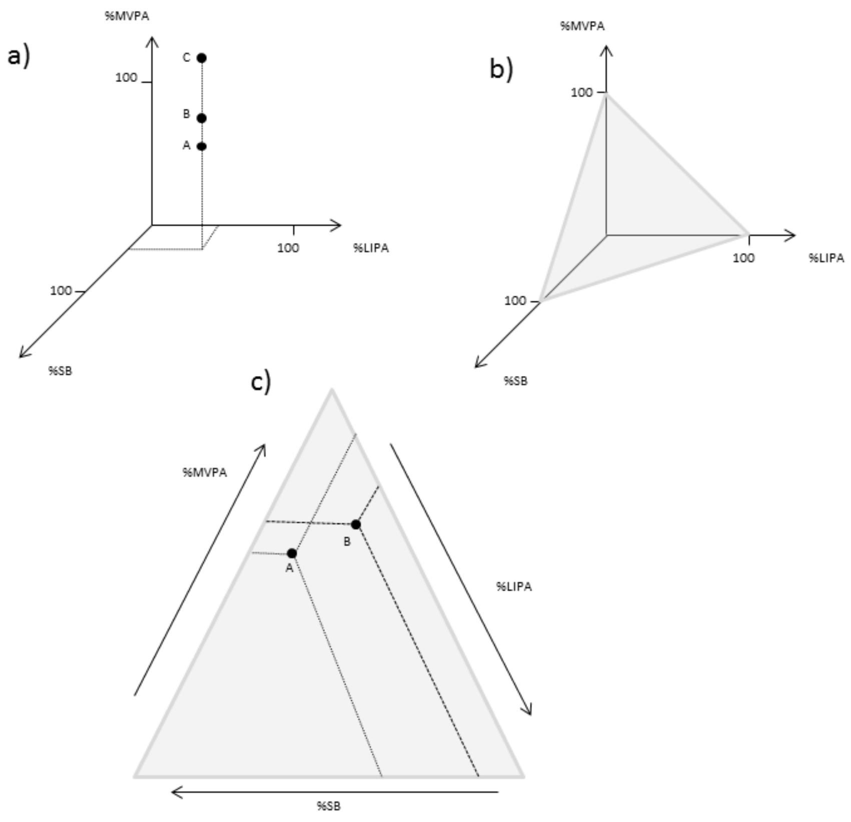

Figure A: a) Data in standard 3-dimensional Cartesian coordinates, b) constrained simplex space occupied by compositional data, c) ternary diagram representation.

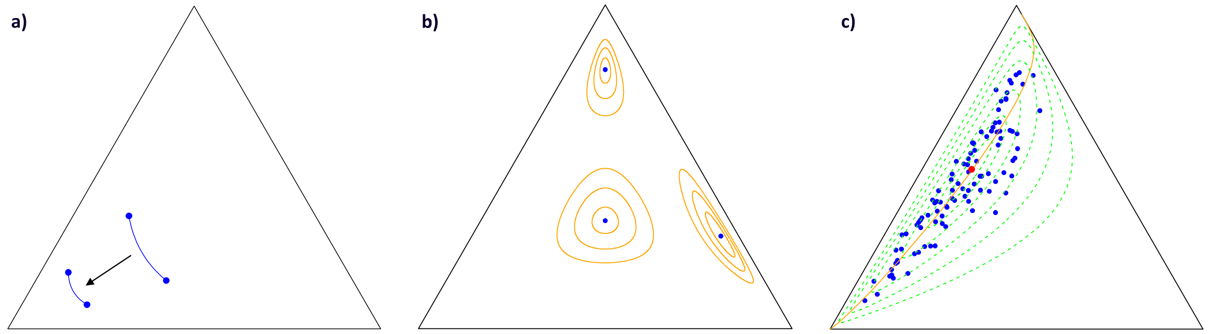

Figure B: Operations and geometrical objects on the simplex: a) translation of points, b) concentric circles, c) regression line and iso-probability ellipses from the center of the cloud of points (big filled point).

Fig. C below shows the 3-part composition of physical activity behavior for adults (aged 21 to 65) from the NHANES 2005-06 data. This plot zooms in the area of interest in the ternary plot. Each dot represents a participant composition as % of time in SB, LIPA and MVPA. The distribution of the sample compositions is depicted as a heat map and the dotted lines give the 90, 95 and 99% normal-

based probability regions for possible compositions in the population.

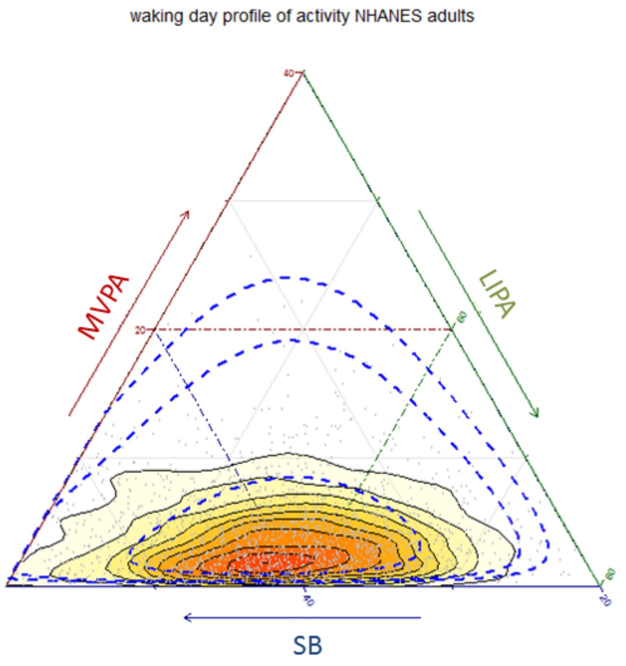

Figure C: Composition of the waking day in terms of percentage of time spent in MVPA, LIPA and SB for adults in the NHANES 2005-6 accelerometry data. Heat map depicts the frequency distribution of compositions and dotted line the 90, 95% and 99% probability regions for possible profile in the population.

A compositional approach implies a change of perspective that focuses on the relative structure of variation of the data. Compositional analysis is about changing how we conceptualize data from the standard real space to the constrained simplex space perspective. This requires that we abandon thinking of each behavior as an independent variable and, instead, view them as relative to the other ones. This implies reasoning in terms of balances between behaviors, as we will see in the following, but also looking at the distribution of data points in ternary plots in a way different to ordinary scatterplots.

DESCRIPTIVE STATISTICS

CENTRAL TENDENCY

It has been shown that the composition best representing the center of a d-part compositional data set is obtained as

$$cen = C(g_1, \dots, g_d)$$

where C refers to the closure operator that close the data in order for them to add up to a constant total. For example, when the data are closed to 1 each part is divided by the sum of all the parts to write them as proportions. By g_i we denote the geometric mean of the ith behavior. Hence, in practice, we first compute the geometric mean of the time spent in each behavior and the resulting vector is then closed to the corresponding constant total according to the scale of the data. This measure of central tendency has been called compositional geometric mean.

DISPERSION

As with confidence intervals, obtaining the variance of a single part is not informative, as it is not considering the co-dependence between parts. In the example above the variance of MVPA is not meaningful as this depends on the variance of SB and LIPA. Moreover, the co-dependence between parts cannot be described by raw correlations or covariances, because they are spurious as a consequence of the closure of the data. In compositional analysis a meaningful estimation of the relative dispersion structure is estimated by what is called the variation matrix, which is a symmetric matrix that contains all the possible log-ratio variances. That is, the variances of the logarithms of all pair-wise ratios between parts. A value close to zero implies that the two parts involved in the ratio (arranged by rows and columns in the matrix) are highly proportional. This is a key change in the way we usually understand co-dependence between variables. In compositional data analysis it is a relation of proportionality. For the adults NHANES data set the variation matrix for waking day behaviors (SB,LIPA and MVPA) is

	SB	LIPA	MVPA
SB	0.0000	0.2484	1.2856
LIPA	0.2484	0.0000	0.9086
MVPA	1.2856	0.9086	0.0000

For example, the variance of $\ln(\text{LIPA}/\text{SB}) = 0.2484$. A variance close to 0 implies that time spent in the corresponding behaviors are nearly proportional, hence, there is a high relationship/co-dependence (in proportionality terms) between them. For example, the co-dependence (proportionality) of one behavior with itself is perfect and, hence, the corresponding log-ratio variance (on the diagonal of the variation matrix) is zero, in an analogous way as the correlation of a variable with itself is one. In our case, the highest log-ratio variances in the matrix both involve MVPA, reflecting a low co-dependence (not low correlation) between this behavior and the others. Note that, in order to help in the interpretation of the variation matrix in terms similar to the ordinary correlation, you can compute $\exp\{-t^2/2\}$, where t is any log-ratio variance. This measure

ranges between 0 and 1, with values close to 1 implying high co-dependence (proportionality) and values close to 0 implying low co-dependence.

RELATIVE BEHAVIOR PROFILES

A composition can be represented in standard barplots, however, in order to appreciate the co-dependence between parts and emphasize the relative differences between subgroups of interest, we can use what can be called a compositional geometric mean barplot by group. We first center the data set by dividing each composition by the overall mean composition given by cen (part by part) and applying the closure operator to the result. This calculation is equivalent to the one usually carried out with ordinary data by subtracting the mean and making the data set to have zero mean. With compositional data the center of the simplex is located at the point where all parts contain the same relative amount. For example, for a 3-behaviour composition, this is the composition given by $cen^0 = (1/3, 1/3, 1/3)$, the barycenter of the triangle. From the centered data, the compositional centers of each group of interest (e.g. different levels of BMI) are calculated separately, denoted by cen_i^0 for a group i. Finally, the log-ratios $\ln(cen_i^0/cen^0)$ are computed for each part and plotted in a barplot, as in Fig. D for the composition (SB, LIPA, MVPA) considering two groups.

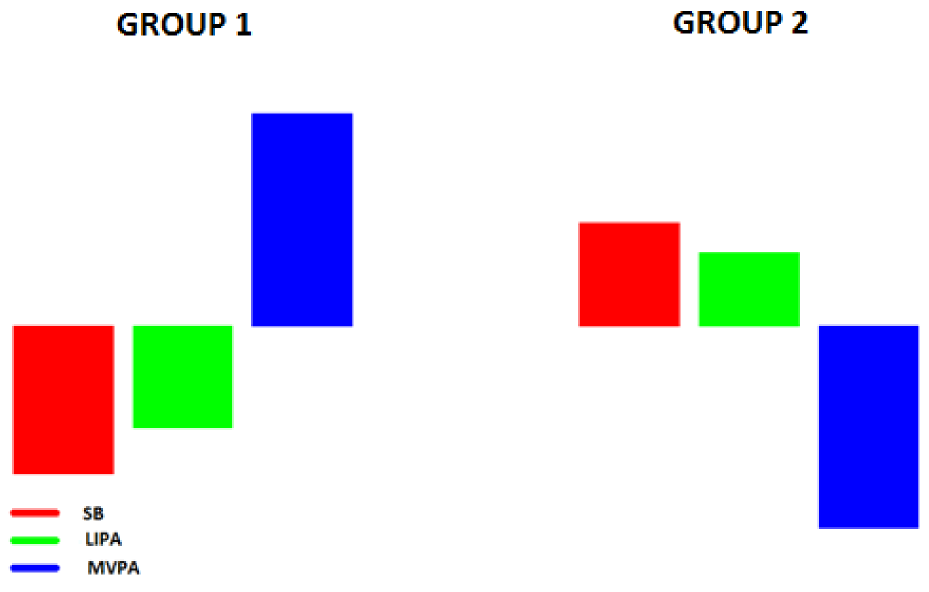

Figure D: Compositional geometric mean barplots for two groups.

In Fig. D each bar is a component of $\ln(cen_i^0/cen^0)$, one per part. Positive and negative bars reflect relative mean values of a part above and below the overall mean composition respectively. This graphical representation is useful to investigate the relative profiles across groups and characterize them. For example, in Fig. D we can see that group 1 is characterized by people spending a relatively high amount of time in MVPA and low in SB and also LIPA. The opposite profile is observed for group 2.

LINEAR REGRESSION WITH COMPOSITIONAL DATA

Compositional data analysis encompasses a vast array of multivariate and graphical statistical techniques, which cannot be described here. A lot of novel exploration of physical behavior data could be achieved with those. Here we focus on linear regression for epidemiology as an entry point and explore ways of obtaining results that can be easily interpreted and compared with the current literature. We describe only a technique that can be implemented easily using standard regression routines and software. Further reading about compositional regression and compositional methods in general can be found in the reference list.

Linear regression analysis with compositional data, where the physical activity composition is acting as explanatory variable and a health outcome is the response variable, follows three steps

1) Transformation of the data.
2) Model fitting using standard estimation procedures.
3) Interpretation of the result and inference within a compositional paradigm using some careful back transformation.

The estimation can be done with standard techniques but the interpretation of the results needs to be done with careful consideration of the compositional nature of the data and using the concept of balances, log-ratios of geometric means, between parts of the composition. Further details can be found in [5].

LOG-RATIO TRANSFORM SETTING OF THE MODEL

In a linear model we try to estimate or predict the value of an outcome Y (conditional expected value) based on the observed time spent on a composition of behaviors B.

If B is made of d parts such as

$B = \sum_{i=1}^{d} b_i$ (1)

the expected value of Y is

$E(Y|B) = \beta_0 + \beta_1 b_1 + \cdots + \beta_d b_d$ (2)

As an example let's consider a partition of the day into three behaviors SB, LIPA and MVPA. In this case we would like to have a model such as

$E(Y|B) = \beta_0 + \beta_1 SB + \beta_2 LIPA + \beta_3 MVPA + covariates$ (3)

We could do this using standard linear regression analysis but this can provide misleading results because of the inherent co-dependence and collinearity between the behaviors. Recall that the standard regression technique assumes unconstrained data on the real space. That is, numerical variables free to vary in $[-\infty, +\infty]$. However, most standard linear regression techniques can be

adapted to compositional data by simply transforming the data to map them from their natural space, the constrained simplex S^d,

$$S^d = \{ B = (b_1, \dots, b_d), \text{with } b_i > 0,\ \sum_{i=1}^{d} b_i = k\} \ (4)$$

with k being the constant total (e.g. 1 when proportions) onto the ordinary real space where those techniques work well. To do this we need to consider log-ratios of time spent in different behaviors rather than the absolute times. In our example

$$S^3 = \{ B = (SB, LIPA, MVPA), \text{with } b_i > 0,\ \sum_{i}^{3} b_i = 100\%\ of\ time\} \ (5)$$

There are different types of log-ratio transformations useful for compositional data analysis. We focus on one type called isometric log-ratio (ilr) transformations. They allow for an isometric mapping (what means that the relative positions of the data points are preserved from the simplex to the real space) between the simplex of d-part compositions and the $(d-1)$-dimensional real space. That is, if the original composition consists of 3 parts, then we obtain 2 ilr-transformed variables. These new variables are real data and as such we can apply standard statistical tools on them. There are infinitely many ilr transformations. One particularly convenient for regression analysis computes the ilr-variables z_i as

$$z_i = \sqrt{\frac{d-i}{d-i+1}} \ln \frac{b_i}{\sqrt[d-i]{\prod_{j=i+1}^{d} b_j}} \text{ with } i = 1, 2, \dots, d-1 \ (6)$$

The linear model (2) can be replaced by

$$E(Y|Z) = \gamma_0 + \gamma_1 z_1 + \gamma_2 z_2 + \gamma_3 z_3 + \cdots + \gamma_{d-1} z_{d-1} + covariates \ (7)$$

where $Z = (z_1, \dots, z_{d-1})$ refers to the vector of all the ilr-variables. This model accounts for all portions of time spent in each (measurable) behavior that add up to a finite time. It therefore accounts for the combined effect of all parts of the composition. It does not matter in what order the parts are transformed, the model gives the same fit with identical R^2, p-value for the model, and coefficient for the intercept γ_0. The interpretation of the common measures associated to the model fitting is the same as for the standard model. The R^2 coefficient tells us how much of the variance is explained by the composition, the p-value for the model tells us if it is a statistically significant model. Interpreting the γ_i coefficients requires more care and will be discussed in the next section.

In the example of a 3-part composition as above, we can write

b_1= SB, b_2= LIPA, b_3 =MVPA and

$$z_1 = \sqrt{\frac{2}{3}} \ln \frac{SB}{\sqrt[2]{LIPA \times MVPA}} \ (8)$$

$$z_2 = \sqrt{\frac{1}{2}} \ln \frac{LIPA}{\sqrt[1]{MVPA}} \ (9)$$

and the model (3) will be

$$E(Y|Z) = \gamma_0 + \gamma_1 z_1 + \gamma_2 z_2 + covariates \ (10)$$

Interpreting the model

As said above, the statistics of models such as (7) or (10) including R^2, p-value for the model, and estimated value of the intercept γ_0, should be interpreted as for any linear model. In particular the model p-value is an indicator of whether or not the composition has a significant association with the outcome Y. In standard linear regression we normally interpret each β_i as the strength of the association between the behavior b_i and Y. This is often used to understand if time spent in a specific behavior has an independent effect on Y and to quantify this potential effect. As discussed above, because of the compositional nature of time spent in physical activity behavior, this way of thinking in largely nonsensical and should be abandoned. Any conclusion drawn from this approach is likely to have limited trustworthiness. Instead we should reason in terms of relative amount spent in one behavior with respect to the others. Below two complementary approaches are detailed.

Interpreting the regression coefficient

It is easy to see that the ilr-transformed variable z_1 explains the ratio between time spent in behavior b_1 and all the others. Therefore γ_1 can be directly interpreted as the strength of the association between the amount of time spent in b_1 relative to the other behaviors and the outcome Y. In model (10), γ_1 is the strength of the association between the relative time spent in SB, compared to LIPA and MVPA, and the outcome. The p-value given for γ_1 can be used as usually to determine whether the behavior b_1 is statistically significant or not to explain the variation in Y. It gives a hint that b_1 is a significant part of the composition for Y. However it should not be interpreted as a sign that b_1 is a predictor of Y independently of the other behaviors, or that b_1 is independently associated with Y. In the example and model (10), if the p-value for γ_1 is smaller than 0.05 (considering the usual 95% confidence level) it indicates that the relative amount of time spent in SB is statistically significantly associated with Y, but it does not mean that SB is an independent predictor of Y.

It is not possible however to interpret z_2, or generally $z_2, \dots, z_{d-1}$, in the same way using the same model. So the question is how to extract information also about $b_2, \dots, b_d$, to obtain the same type of information for LIPA as we do for SB in model (10) above.

Because of the permutation principle, the regression model with d parts will give the same fit regardless of the order of them. It is therefore possible to construct d equivalent models with each behavior sequentially playing the role of first part of the composition and being transformed into z_1, and then interpret each γ_1 and associated p-value sequentially.

In the context of the 3-part example above, we can write 3 models.

Model 1 $E^1(Y|Z) = \gamma_0 + \gamma_1 {}^1z_1{}^1 + \gamma_2 {}^1z_2{}^1 + covariates$ (11)

with b_1= SB, b_2= LIPA, b_3 = MVPA and

$$z_1 = \sqrt{\frac{2}{3}} \ln \frac{SB}{\sqrt[2]{LIPA \times MVPA}}$$

$$z_2 = \sqrt{\frac{1}{2}} \ln \frac{LIPA}{\sqrt[1]{MVPA}}$$

Model 2 $E^2(Y|Z) = \gamma_0 + \gamma_1{}^2 z_1{}^2 + \gamma_2{}^2 z_2{}^2 + covariates$ (12)

with b_1= LIPA, b_2= MVPA, b_3 = SB and

$$z_1 = \sqrt{\frac{2}{3}} \ln \frac{LIPA}{\sqrt[2]{SB \times MVPA}}$$

$$z_2 = \sqrt{\frac{1}{2}} \ln \frac{MVPA}{\sqrt[1]{SB}}$$

Model 3 $E^3(Y|Z) = \gamma_0 + \gamma_1{}^3 z_1{}^3 + \gamma_2{}^3 z_2{}^3 + covariates$ (13)

with b_1= MVPA, b_2= SB, b_3 = LIPA and

$$z_1 = \sqrt{\frac{2}{3}} \ln \frac{MVPA}{\sqrt[2]{SB \times LIPA}}$$

$$z_2 = \sqrt{\frac{1}{2}} \ln \frac{SB}{\sqrt[1]{LIPA}}$$

We then need to interpret $\gamma_1{}^1$ from (11) which gives information about the association of the relative amount of time spent in SB as before, $\gamma_1{}^2$ from (12) which gives the same information for LIPA and, finally, $\gamma_1{}^3$ in relation to MVPA.

Note that, the R^2, the p-value for the model, the estimated value for the intercept γ_0 and all the covariates should be the same for all these three models (11-13). This is actually a good way to check that no mistakes were made during the sequential permutation.

QUANTIFYING THE EFFECT SIZE

In standard regression the coefficients β_i are directly interpreted as the change in Y associated with a change in b_i. For compositional data this is not the case because a change in b_i is necessarily also concomitant with a change in the other behaviors. Moreover, the coefficients γ_i associated to the log-ratios z_i are not easily interpretable in terms of units of change of the raw behaviors.

However, it is possible to quantify the effect of changing b_i by considering log-ratios between time spent in b_i and the other behaviors. This has the advantage of, not only giving a quantification of the effect of changing time spent in b_i, but also quantifying this depending on which other behavior this change in b_i is displacing. This can be achieved by computing a change prediction matrix as follows. This matrix can be obtained by firstly applying the inverse ilr transformation to the coefficients $\gamma_1, \dots, \gamma_{d-1}$ of the linear model (7) to obtain the composition $U = (u_1, \dots., u_d)$ associated to them in the simplex. The change matrix is then given by

	b_1	b_2	….	b_d
b_1	0	$c_{12} = \frac{ln(u_1/u_2)}{4d^2}$	….	$c_{1p} = \frac{ln(u_1/u_d)}{4d^2}$
b_2	$c_{21} = \frac{ln(u_2/u_1)}{4d^2}$	0	….	$c_{2p} = \frac{ln(u_2/u_d)}{4d^2}$
….	….	….	0	….
b_d	$c_{p1} = \frac{ln(u_d/u_1)}{4d^2}$	$c_{p2} = \frac{ln(u_d/u_2)}{4d^2}$	….	0

The value c_{ij} in the matrix corresponds to change in Y in response to change in the ratio between the times spent in behaviors b_i and b_j by the mathematical constant e, the reciprocal of the natural logarithm ln, which is approximately equal to 2.718. If c_{ij} is positive the change corresponds to an increase in Y and if it is negative the change corresponds to a decrease. This is in a sense similar to isotemporal substitution, but considering all possible substitutions between behaviors in the composition of the day.

It is possible to obtain more directly relevant and interpretable c_{ij} values by considering a unit change in behavior b_i. For example, we could evaluate the effect of substituting 10 minutes, and computing the change in log-ratio for all pairs of behaviors around a set point, say the mean composition. Multiplying the change in the ratio b_i/b_j by c_{ij} and dividing by e gives the estimated effect on Y of substituting 10 minutes of b_j for b_i. Numerical examples of this are found in the main manuscript.

CONFIDENCE INTERVALS ON PREDICTION AND GRAPHICAL TECHNIQUE

Usually in linear regression we estimate confidence intervals for the expected value of the outcome predicted by the model given a set of behaviors and adjusted for covariates. Predicted means and associated confidence intervals are often used to summarize the effect of the explanatory variables on the outcome. However, only the change in one behavior is considered, without taking into account the fact that time spent in this behavior necessarily is taken away from another. For example, if we compute the effect of MVPA and find it to be 10 +/- 5 for an outcome, this does not take into account whether this confidence interval corresponds to a change in MVPA from LIPA or from SB. It is possible that the effect on an outcome of increasing MVPA by replacing LIPA or SB is not the same.

Predicted means are typically computed by using the model to predict the outcome over a reference grid of values of the explanatory variables. In order to adapt this to compositional data, the first step is to compute a grid of points in the simplex space. Then, use the ilr transformation to transform this grid into a prediction grid on real space and, finally, predict the outcome using model (7) over this grid. The results can then be plotted as in Fig. E using heat map overlaid on a ternary plot which shows the predicted outcome for different compositions.

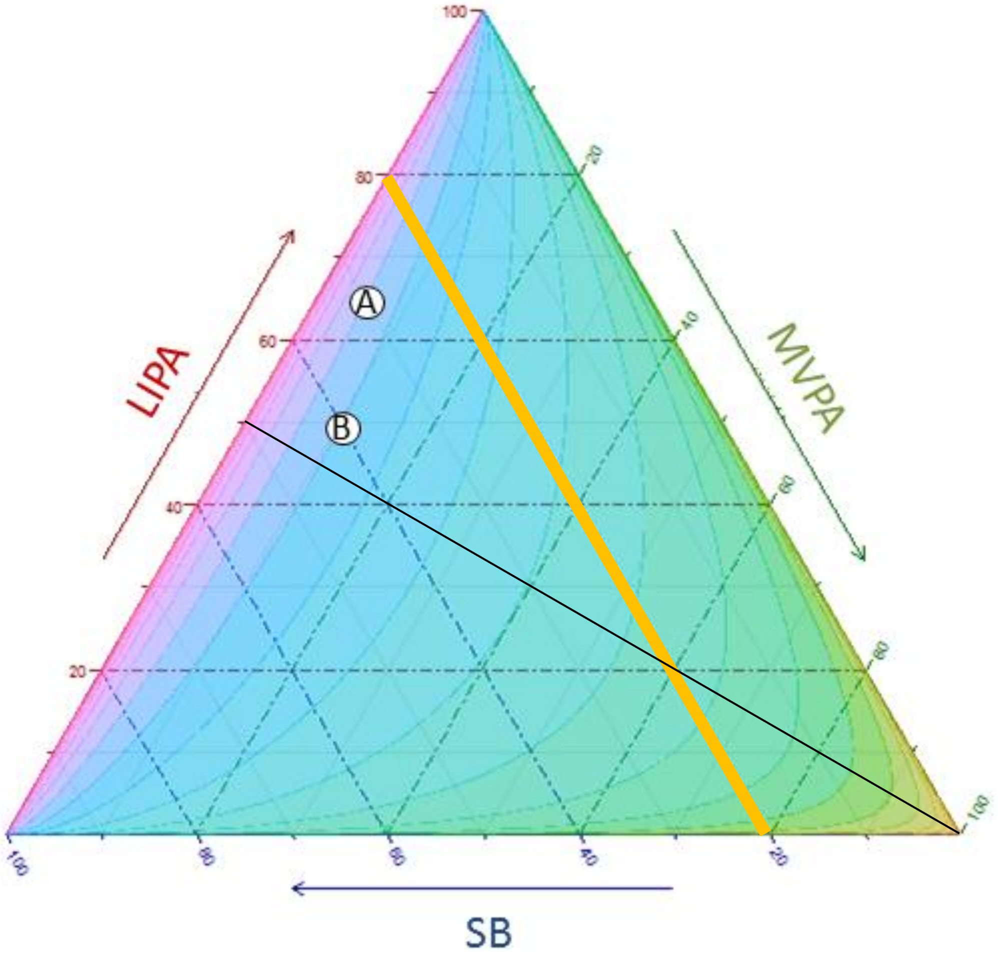

Figure E: Predicted health outcome (here waist circumference) on a ternary heat map (red high, green low).

This enables to identify compositions of time spent in different behaviors associated to likely healthy or unhealthy outcomes. For example point A, which corresponds to a 5% MVPA, 65% LIPA and 30% SB composition is associated with a higher waist circumference (WC) than point B which corresponds to a composition made of 10% MVPA, 50% LIPA and 40% SB.

These predictions, along with 95% confidence intervals at each point, can also be represented in bivariate plots considering pairs of behaviors as in Fig. F. Fig. Fa shows the effect on outcome WC of substituting relative time spent on MVPA for LIPA at different fixed values of SB. Similarly Fig. Fb shows the effect of changing time from LIPA to MVPA given fixed values of SB. The red curves in Fig. Fa and Fb correspond to the outcome along the solid orange line in Fig. E.

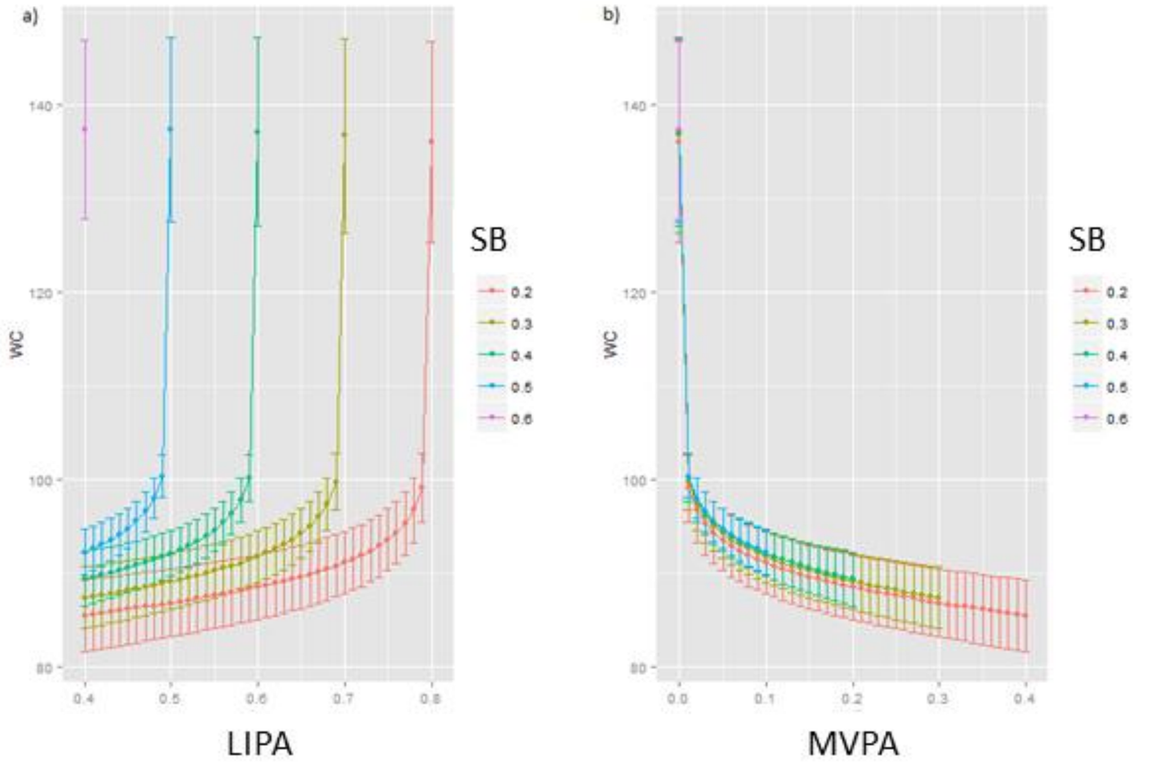

Figure F: Bivariate plots of the effect on waist circumference (WC) of changes in the relative amount of time spent on LIPA and MVPA at fixed relative amounts of SB. They correspond to substituting MVPA for LIPA (graph a) on the left-hand side) and vice versa (graph b) on the right-hand side).

EFFECT OF SUBGROUPS OF PARTS OF THE COMPOSITION

In some cases it may be of interest to investigate whether some behaviors, or time spent on a specific subgroup of behaviors, are more influential on the outcome than others. If we examine the time spent on a large number of sub-behaviors it may be of interest to focus on those having the greatest impact. The p-value of the ilr-variables from the linear models can be a guide giving a hint, but do not offer a definitive answer. A systematic exploration of all possible sub-compositions is recommended. Examining the change matrix introduced before will already give a more tangible and trustworthy estimation of which pairs of behaviors are worth considering. High values of c_{ij} indeed indicate that the two behaviors b_i and b_j are important as a ratio.

The graphics above can also be used to focus the analysis. For example, in Fig. E the predicted outcome appears symmetric along the solid black line. This hints that replacing MVPA with either LIPA or SB has a very similar effect. In this case we may want to consider a new composition including only two behaviors MVPA and (LIPA+SB). Moreover, in Fig. F it is easy to see that replacing MVPA for LIPA as a different effect depending on the proportion of time spent in SB (Fig. Fa). However, on the contrary, the effect of replacing LIPA by MVPA appears to be quite similar for any proportion of SB. This also hints that a partition in two behaviors may be more appropriate for this outcome.

DEALING WITH ZEROS

Compositional techniques rely heavily on log-ratios, hence if a part of the composition is zero this creates a problem as the logarithm is not defined. So, before conducting a compositional analysis it is convenient to check for the presence of zero parts. There are a number of ways to deal with zeros depending on their nature. For example, it is not the same a zero that can be attributable to rounding-off effects, the limitations of the sampling process or to values falling below a certain measurement threshold, than genuine zeros that can characterize a particular subset of individuals. We leave this issue as beyond the scope of this introductory guide and refer the reader to e.g. for more details. The simplest way in practice would be to carefully select an aggregation of parts that is meaningful and does not contain zeros.

REFERENCES

1. Zeljko Pedisic. Measurement issues and poor adjustments for physical activity and sleep undermine sedentary behaviour research. Kinesiology. 2014;46: 135–146.

2. Pearson K. Mathematical contributions to the theory of evolution. On a form of spurious correlation which may arise when indices are used in the measurement of organs. Proceedings of the Royal Society of London,LX. 1897. pp. 489–502.

3. Aitchinson J. The Statistical Analysis of Compositional Data [Internet]. London: Blackburn Press; 2003. Available: http://www.amazon.co.uk/The-Statistical-Analysis-Compositional-Data/dp/1930665784

4. Pawlowsky-Glahn V, Buccianti A. Compositional Data Analysis: Theory and Applications. Chichester: John Wiley and Sons LtD; 2011.

5. Hron K, Filzmoser P, Thompson K. Linear regression with compositional explanatory variables. J Appl Stat. Taylor & Francis; 2012;39: 1115–1128. doi:10.1080/02664763.2011.644268

6. Martín-Fernández JA, Palarea-Albaladejo J, Olea R. Dealing with zeros. In: Pawlowsky-Glahn V, Buccianti A, editors. Compos Data Anal Theory Appl. Chichester: John Wiley & Sons, Ltd; 2011. pp. 43–58.

7. Palarea-Albaladejo J, Martín-Fernández JA. zCompositions — R package for multivariate imputation of left-censored data under a compositional approach. Chemom Intell Lab Syst. 2015;143: 85–96. doi:10.1016/j.chemolab.2015.02.019
